# Proposal for a unified nomenclature for target‐site mutations associated with resistance to fungicides

**DOI:** 10.1002/ps.4301

**Published:** 2016-06-16

**Authors:** Wesley Mair, Francisco Lopez‐Ruiz, Gerd Stammler, William Clark, Fiona Burnett, Derek Hollomon, Hideo Ishii, Tarlochan S Thind, James KM Brown, Bart Fraaije, Hans Cools, Michael Shaw, Sabine Fillinger, Anne-Sophie Walker, Emilia Mellado, Guido Schnabel, Andreas Mehl, Richard P Oliver

**Affiliations:** ^1^ Centre for Crop Disease Management, Department of Environment and AgricultureCurtin UniversityBentleyWAAustralia; ^2^ BASF SE, Agricultural Centre, Fungicide Resistance ResearchLimburgerhofGermany; ^3^ NIABCambridgeUK; ^4^ SRUCEdinburghUK; ^5^ Orchard HouseBristol RoadChew StokeBristolUK; ^6^ School of Agricultural Regional VitalisationKibi International UniversityMinami‐awajiHyogoJapan; ^7^ Punjab Agricultural UniversityLudhianaIndia; ^8^ John Innes CentreNorwichUK; ^9^ Rothamsted ResearchHarpendenHertfordshireUK; ^10^ Syngenta, Jealott's Hill International Research CentreBracknellBerkshireUK; ^11^ University of ReadingReadingBerkshireUK; ^12^ INRAThiverval‐GrignonFrance; ^13^ Centro National de MicrobiologiaInstituto de Salud Carlos III, MajadahondaMadridSpain; ^14^ Department of Agricultural and Environmental SciencesClemson UniversityClemsonSCUSA; ^15^ Bayer CropScience AG, Research Disease ControlMonheimGermany

**Keywords:** fungicide, target site, mutation, SDHI, Cyp51

## Abstract

Evolved resistance to fungicides is a major problem limiting our ability to control agricultural, medical and veterinary pathogens and is frequently associated with substitutions in the amino acid sequence of the target protein. The convention for describing amino acid substitutions is to cite the wild‐type amino acid, the codon number and the new amino acid, using the one‐letter amino acid code. It has frequently been observed that orthologous amino acid mutations have been selected in different species by fungicides from the same mode of action class, but the amino acids have different numbers. These differences in numbering arise from the different lengths of the proteins in each species. The purpose of the present paper is to propose a system for unifying the labelling of amino acids in fungicide target proteins. To do this we have produced alignments between fungicide target proteins of relevant species fitted to a well‐studied ‘archetype’ species. Orthologous amino acids in all species are then assigned numerical ‘labels’ based on the position of the amino acid in the archetype protein. © 2016 The Authors. *Pest Management Science* published by John Wiley & Sons Ltd on behalf of Society of Chemical Industry.

## 1 BACKGROUND

Evolved resistance to fungicides is a major problem limiting our ability to control agricultural, medical and veterinary pathogens.^1^, ^2^ Research over the last 30 years has often defined the mechanism conferring reduced sensitivity to the fungicide. Many cases of resistance have been ascribed to the activity of efflux pumps^3^ or to overexpression of target genes,^4^ but the majority are due, at least partly, to substitutions (or indels) in the amino acid sequence of the target protein.

The convention for describing amino acid substitutions is to cite the wild‐type amino acid, the codon number and the new amino acid, using the one‐letter amino acid code (see Oliver and Hewitt,^5^ box 6.1, p. 138 for a description of the system). A well‐known example is the alanine (A) for glycine (G) substitution in the cytochrome b gene at position 143 conferring resistance to strobilurin fungicides, referred to as G143A.^6^ Further alterations can be amino acid deletions designated with a Δ and insertions with an ‘ins’.

Target‐site amino acid substitutions have been described for seven fungicide groups (named here according to the FRAC convention^7^) and their target proteins. These are C3 and cytochrome b (Cytb) (Table 1); G1 and two sterol C14‐demethylases (paralogues Cyp51A and Cyp51B) (Tables 2 and 3); B1/B2 and b‐tubulin (Table 4); C2 and three of the subunits of the succinate dehydrogenase complex (SdhB, SdhC and SdhD) (Tables 5, 6 and 7); H5 and cellulose synthase A3 (CesA3) (Table 8); E3 and the Os1 family (group III) histidine kinase (Os‐1, includes Bos1, BcOS1, Daf1, HK1, HIK1 and NIK1^8^) (Table 9); G3 and the 3‐keto reductase (Erg27). Where more than one species has been studied, it has frequently been observed that orthologous amino acid mutations have been associated with resistance to fungicides with the same mode of action.

**Table 1:** CytB. Position number based on alignment to reference sequence from Zymoseptoria tritici (NCBI gene accession number AY247413)

Amino acid substitution(s) in archetype	Homologous position in other species
F129L	F129L in PHAKPA
	F129L in PLASVI
	F129L in PYRIOR
	F129L in PYRNTE
	F129L in PYRNTR
	F129L in RHIZSO
G137	G137R in PYRNTR
G143A	G143A in ALTEAL
	G143A in ALTELY
	G143A in ALTESO
	G143A in ALTETO
	G143A in BOTRCI
	G143A in CERCBE
	G143A in COLLGR
	G143A in ERYSGT
	G143A in LEPTNO
	G143A in MICDMA
	G143A in MONGNI
	G143A in MYCOFI
	G143A in MYCORA
	G143A in PLASVI
	G143A in PLEOAL
	G143A in PODOFU
	G143A in PSPECU
	G143A in PYRIOR
	G143A in PYRNTR
	G143A in RHIZSO
	G143A in VENTIN

**Table 2:** Cyp51A. Position number based on alignment to reference sequence from Aspergillus fumigatus (Cyp51A) (NCBI gene accession number AF338659)

Amino acid substitution(s) in archetype	Homologous position in other species
N22D	NA
S52T	NA
G54E/K/R/V/W	G54W in ASPEPA
Y68	Y132N in ASPEFL
Q88H	NA
L98H	NA
V101F	NA
Y121F	Y136F in AJELCP
N125I	NA
K133	K197N in ASPEFL
G138C/R/S	NA
Q141H	NA
H147Y	NA
P216L	NA
F219S	NA
M220K/I/T/V	NA
D280	D282E in ASPEFL
M286	M288L in ASPEFL
T289A	NA
S297T	NA
P394L	NA
Y431C	NA
G432S	NA
G434C	NA
T440A	NA
G448S	NA
T470	T469S in ASPEFL
Y491H	NA
F495I	NA

**Table 3:** Cyp51B. Position number based on alignment to reference sequence from Zymoseptoria tritici (NCBI gene accession number AY253234)

Amino acid substitution(s) in archetype	Homologous position in other species
T66	A61V in CANDAL
C80	S79T in ERYSGT
D107V	NA
L126	F120L in PHAKPA
D134G	NA
V136A/C/G	NA
Y137F	Y132F/H in CANDAL
	Y131F/H in PHAKPA
	Y134F in PUCCRT
	Y136F in ERYSGH
	Y136F in ERYSGT
	Y136F in MONIFC
	Y136F in MYCOFI
	Y136F in UNCINE
	Y140F/H in SACCCE
	Y145F in FILBNF
M145L	NA
K148	K142R in PHAKPA
	K143E in CANDAL
	K147Q in ERYSGH
V151	I145F in PHAKPA
D176	K175N in ERYSGT
N178S	NA
S208T	NA
N284H	NA
E300	E297K in CERCBE
H303Y	NA
A311G	A313G in MYCOFI
G312A	NA
I333	I330T in CERCBE
A379G	A381G in MYCOFI
I381V	NA
P391	P384S in CERCBE
A410T	S405F in CANDAL
G412A	NA
H430	H399P in ASPEFL
A453	D411N in ASPEFL
Y459C/D/N/S/P/Δ	Y461D in MYCOFI
G460D/Δ	G462A in MYCOFI
Y461D/H/S	F449S in CANDAL
	Y463D/H/N in MYCOFI
G476	G464S in CANDAL
	G484S in FILBNF
R479	R467K in CANDAL
I483	I471T in CANDAL
	I475T in PHAKPA
V490L	NA
T496	T454P in ASPEFL
G510C	NA
S524T	S508T in PYRPBR
	S509T in ERYSGH

**Table 4:** b‐Tubulin. Position number based on alignment to reference sequence from Aspergillus nidulans (benA) (NCBI gene accession number M17519)

Amino acid substitution(s) in archetype	Homologous position in other species
H6L/Y	H6Y in LEPTNO
	H6Y in MONIFC
Y50N/S	Y50N in GIBBFU β_1_‐tubulin
	Y50C in GIBBZE β_2_‐tubulin
	Y50C in HYPMOD
M73	Q73R in GIBBZE β_2_‐tubulin
Q134K	NA
A165V	NA
F167	F167Y in CERCBE
	F167Y in COCHHE
	F167Y in GIBBZE β_2_‐tubulin
	F167Y in NEUSCR
	F167Y in PENIEX
E198D/K/Q	E198A/G/K/V in BOTRCI
	E198A in CERCBE
	E198V in GIBBFU β_2_‐tubulin
	E198K/L/Q in GIBBZE β_2_‐tubulin
	E198A/Q in HELMSO
	E198A/K in MONIFC
	E198G in NEUSCR
	E198A/K in PENIAU
	E198A/K/V in PENIEX
	E198K in PENIIT
	E198A/G in PYRPBR
	E198G/K in RHYNSE
	E198A/K in SCLEHO
	E198A in SCLESC
	E198A/K in VENTIN
F200Y	F200Y in BOTRCI
	F200Y in GIBBFU β_2_‐tubulin
	F200Y in GIBBZE β_2_‐tubulin
	F200Y in PENIAU
	F200Y in PENIIT
	F200Y in RHYNSE
	F200Y in VENTIN
L240	L240F in MONILA
	L240F in PYRPBR
	L240F in VENTIN
M257L	NA

**Table 5:** SdhB. Position number based on alignment to reference sequence from Pyrenophora teres f. sp. teres (NCBI gene accession number XM_003302513)

Amino acid substitution(s) in archetype	Homologous position in other species
P230	P225F/L/T in BOTRCI
N235	N225I/T in SEPTTR
	N230I in BOTRCI
H277Y	H249L/N/Y in EUROOR
	H257L in USTIMA
	H267L/R/Y in SEPTTR
	H273Y in SCLESC
	H272L/R/V/Y in BOTRCI
	H277R/Y in ALTEAL
	H277R/Y in ALTESO
	H277R/Y in DIDYBR
	H278R/Y in CORYCA
	H → Y in PODOXA^a^
I279	I269V in SEPTTR

a The amino acid position number for this substitution is unknown as only a 176 bp fragment of SdhB gene has been sequenced in both sensitive and resistant isolates.

**Table 6:** SdhC. Position number based on alignment to reference sequence from Pyrenophora teres f. sp. teres (NCBI gene accession number XM_003302752)

Amino acid substitution(s) in archetype	Homologous position in other species
T68	T79I/N in SEPTTR
W69	W80S in SEPTTR
S73	S73P in CORYCA
	A84V in SEPTTR
	A85V in BOTRCI
N75S	N86K/S in SEPTTR
T78	T90I in EUROOR
G79R	G90R in SEPTTR
H134R	H134R in ALTEAL
	H146R in SCLESC
S135R	NA
H141	H152R in SEPTTR

**Table 7:** SdhD. Position number based on alignment to reference sequence from Pyrenophora teres f. sp. teres (NCBI gene accession number XM_003297196)

Amino acid substitution(s) in archetype	Homologous position in other species
S118	S89P in CORYCA
D124E/N	NA
H134R	H132R in BOTRCI
	H132R in SCLESC
	H133R in ALTEAL
	H133R in ALTESO
G138	G109V in CORYCA
D145G	D124E in EUROOR
	D129E in SEPTTR

**Table 8:** CesA3. Position number based on alignment to reference sequence from Phytophthora infestans (NCBI gene accession number EF563995)

Amino acid substitution(s) in archetype	Homologous position in other species
Q1077	Q1077K in PHYTCP
G1105A/V	G1105S/V in PLASVI
	G1105V/W in PSPECU
V1109L	V1109L/M in PHYTCP
	V1109L in PHYTDR

**Table 9:** OS‐1. Position number based on alignment to reference sequence from Botrytis cinerea (Bos1) (NCBI gene accession number AF435964)

Amino acid substitution(s) in archetype	Homologous position in other species
F250	F267L in PLEOAL
I273	L290S in PLEOAL
I365N/R/S	NA
V368F	NA
Q369H/P	NA
N373S	NA
G403	G420D in ALTELO
T447S	NA
E738	E753K in ALTEBI
T750	T765R in PLEOAL
Q762	Q777R in PLEOAL

In cases where the proteins are strongly conserved between species, the mutations have identical numbers. For example, the orthologous Cytb G143A mutation has been found in 22 species (Table 1). However in other cases orthologous mutations have different numbers – e.g. Cyp51B amino acid Y137 in Zymoseptoria tritici is orthologous to amino acids numbered from 131 to 145 in different species (Table 3). Similarly, SdhB amino acid H277 in Pyrenophora teres is orthologous to amino acids numbered from 249 to 278 (Table 5). These differences in numbers create unnecessary confusion and obscure the relationships between mutations in different species.

Resistance caused by insertions in promoters and in efflux pumps have a much lower level of homology and so are not considered here.

## 2 THE PROPOSAL

The differences in numbering arise from the different lengths of the fungicide target protein in each species. The purpose of the present paper is to propose a system for unifying the labelling of mutant amino acids in fungicide target proteins. We propose that orthologous amino acids (i.e. ones presumed to be descended from the same amino acid in the common ancestor of these species) are given the same number in all species regardless of the actual position. The advantages of a unified system is that it would be easier to memorise common changes, to determine whether the changes were novel or were repetitions of what has already been seen in other species and to link changes to particular active ingredients. Orthologous mutations would be assigned the same ‘mutation label’.

We distinguish between ‘mutation labels’, which refer to the orthology between proteins from different species, and ‘amino acid numbering’, which remains the order of the amino acids in each protein in each species. To avoid confusion, we propose that mutation labels should be italicised and mutation numbers should use regular lettering.

In several cases, amino acid substitutions have been found in the target protein but have not been definitively associated with any change in sensitivity either in vitro or in the field. It may be that the mutation underlying the amino acid substitution is a random event and of no obvious relevance. Definitively linking a mutation to a sensitivity change can be technically very demanding. If resistance to the same class of fungicide is linked to mutations affecting orthologous codons in different species, this is strong, if still circumstantial, evidence of the importance of the mutation. Unifying the mutant labelling system will make it much easier to identify important codon changes. This would assist the prioritisation of research aiming functionally to characterise mutations.

## 3 OPTIONS FOR PRODUCING THE ALIGNMENTS

**Figure 1:**
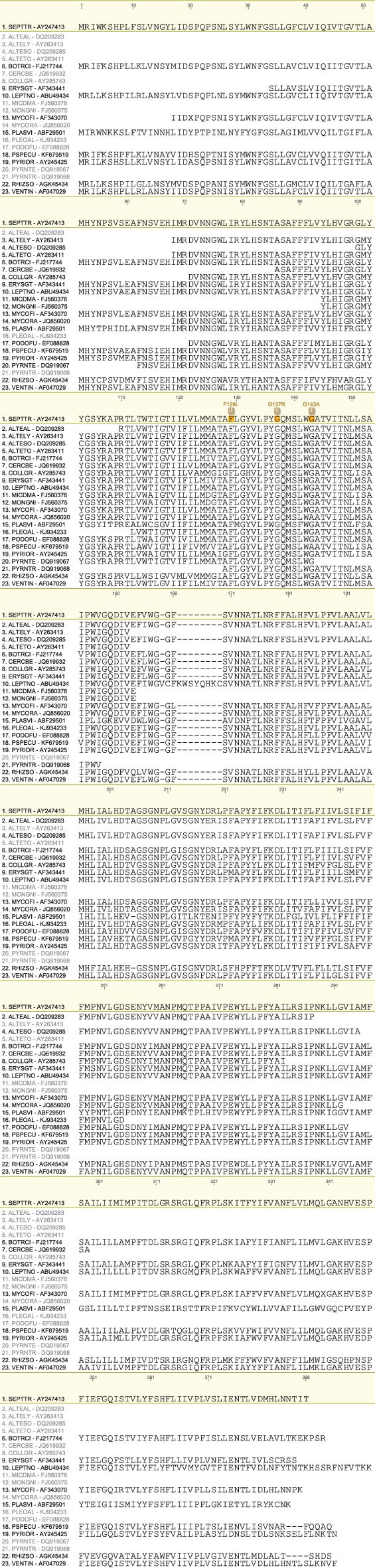
Amino acid sequence alignment of the Cytb family. Sequences are named by species EPPO code and NCBI gene accession number. Residues highlighted in yellow in the archetype sequence from Zymoseptoria tritici denote amino acid substitutions associated with fungicide resistance at an orthologous position in any of the sequences. Numerical mutation labels shown above the alignment are based on the position number of the amino acid in the archetype protein.

**Figure 2:**
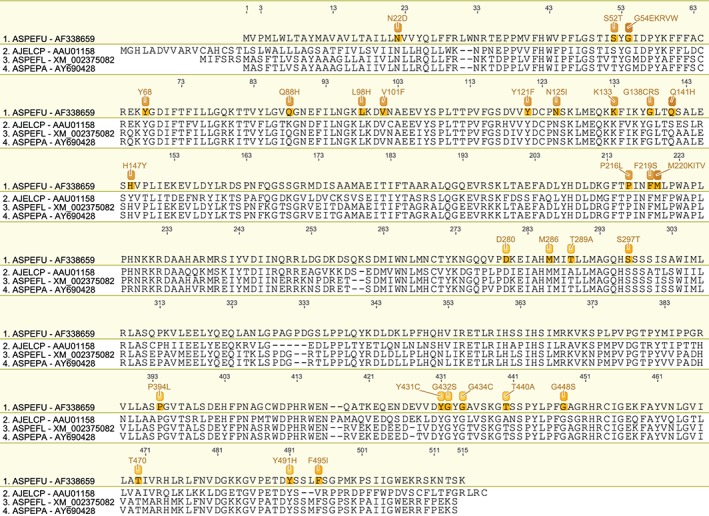
Amino acid sequence alignment of the Cyp51A family. Sequences are named by species EPPO code and NCBI gene accession number. Residues highlighted in yellow in the archetype sequence from Aspergillus fumigatus (Cyp51A) denote amino acid substitutions associated with fungicide resistance at an orthologous position in any of the sequences. Numerical mutation labels shown above the alignment are based on the position number of the amino acid in the archetype protein.

**Figure 3:**
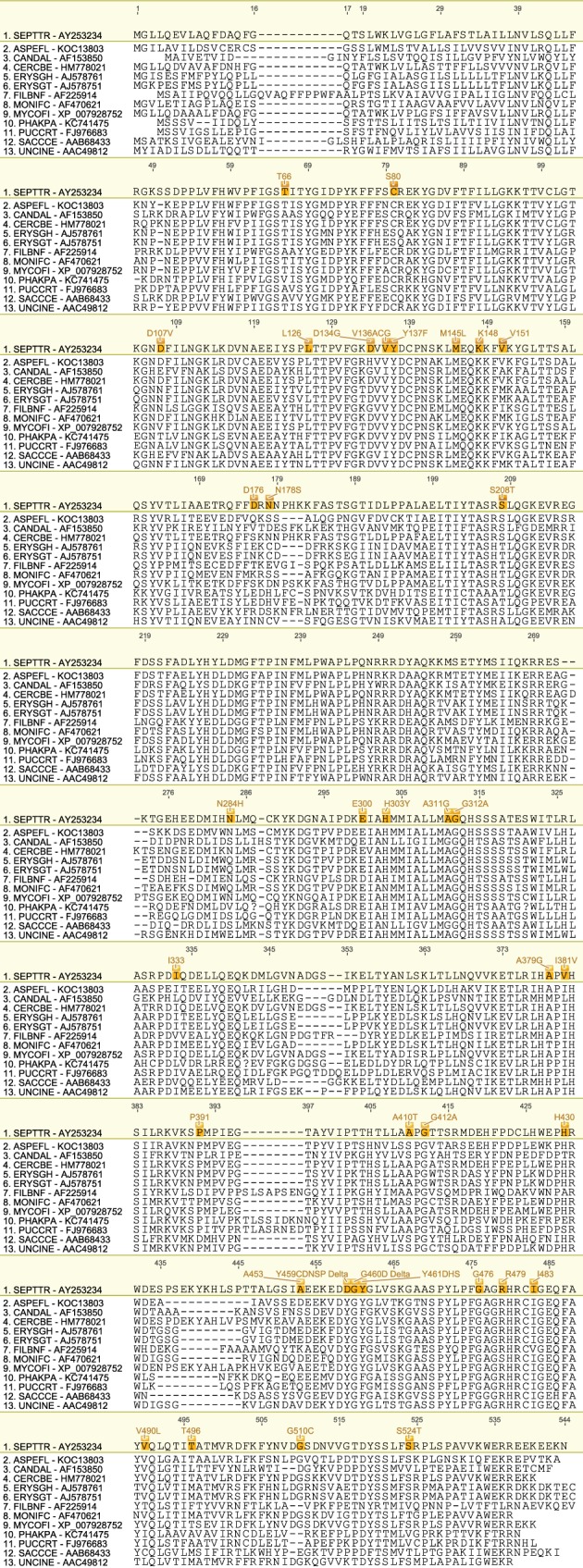
Amino acid sequence alignment of the Cyp51B family. Sequences are named by species EPPO code and NCBI gene accession number. Residues highlighted in yellow in the archetype sequence from Zymoseptoria tritici denote amino acid substitutions associated with fungicide resistance at an orthologous position in any of the sequences. Numerical mutation labels shown above the alignment are based on the position number of the amino acid in the archetype protein.

**Figure 4:**
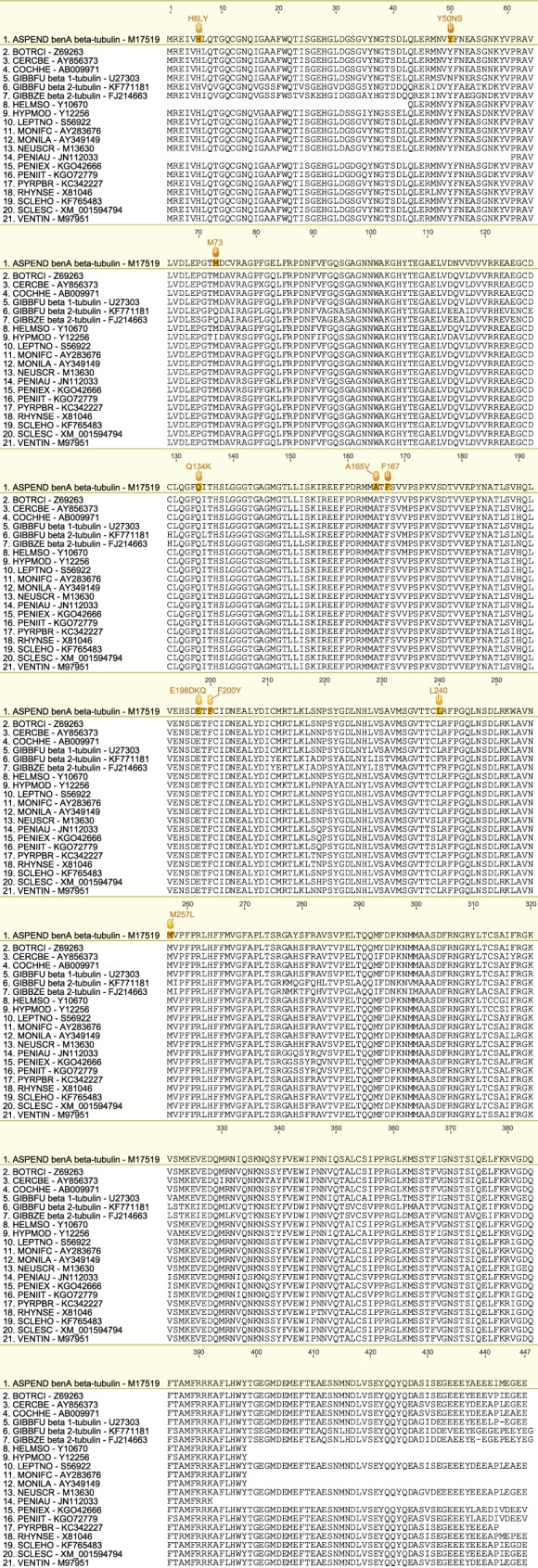
Amino acid sequence alignment of the b‐tubulin family. Sequences are named by species EPPO code and NCBI gene accession number. Residues highlighted in yellow in the archetype sequence from Aspergillus nidulans (benA) denote amino acid substitutions associated with fungicide resistance at an orthologous position in any of the sequences. Numerical mutation labels shown above the alignment are based on the position number of the amino acid in the archetype protein.

**Figure 5:**
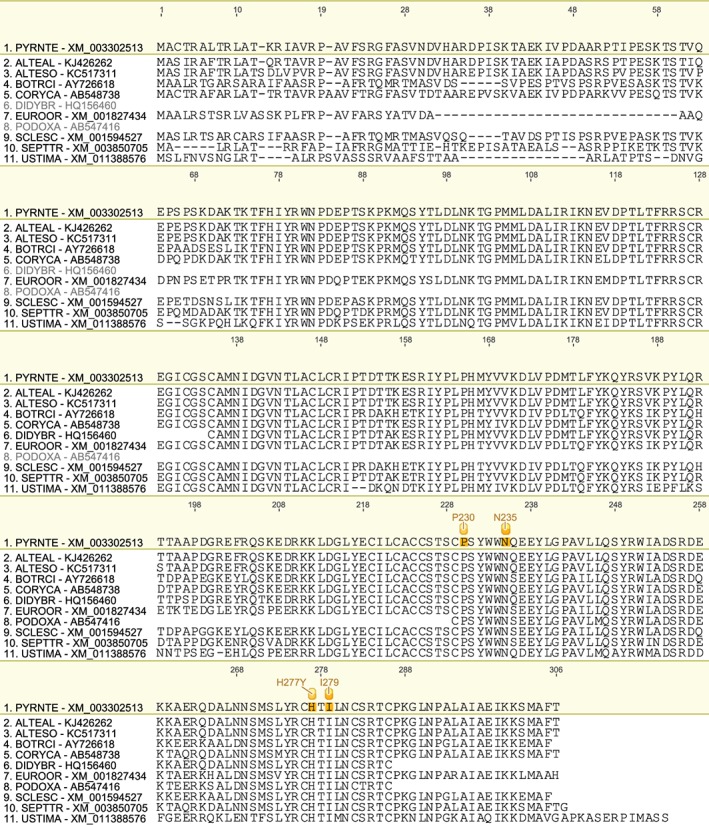
Amino acid sequence alignment of the SdhB family. Sequences are named by species EPPO code and NCBI gene accession number. Residues highlighted in yellow in the archetype sequence from Pyrenophora teres f. sp. teres denote amino acid substitutions associated with fungicide resistance at an orthologous position in any of the sequences. Numerical mutation labels shown above the alignment are based on the position number of the amino acid in the archetype protein.

**Figure 6:**
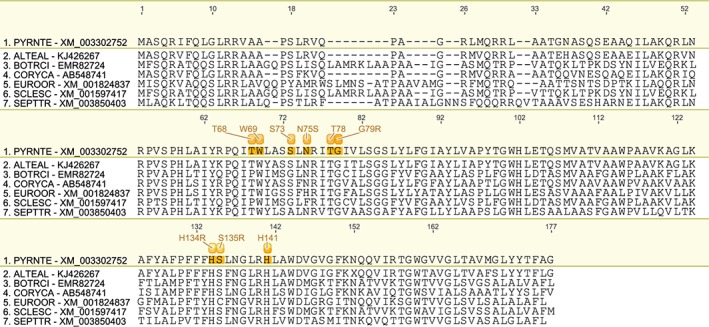
Amino acid sequence alignment of the SdhC family. Sequences are named by species EPPO code and NCBI gene accession number. Residues highlighted in yellow in the archetype sequence from Pyrenophora teres f. sp. teres denote amino acid substitutions associated with fungicide resistance at an orthologous position in any of the sequences. Numerical mutation labels shown above the alignment are based on the position number of the amino acid in the archetype protein.

**Figure 7:**
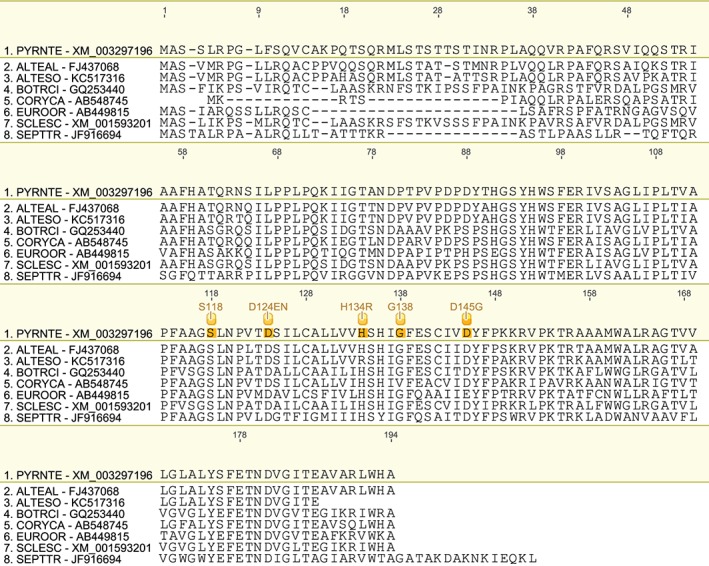
Amino acid sequence alignment of the SdhD family. Sequences are named by species EPPO code and NCBI gene accession number. Residues highlighted in yellow in the archetype sequence from Pyrenophora teres f. sp. teres denote amino acid substitutions associated with fungicide resistance at an orthologous position in any of the sequences. Numerical mutation labels shown above the alignment are based on the position number of the amino acid in the archetype protein.

**Figure 8:**
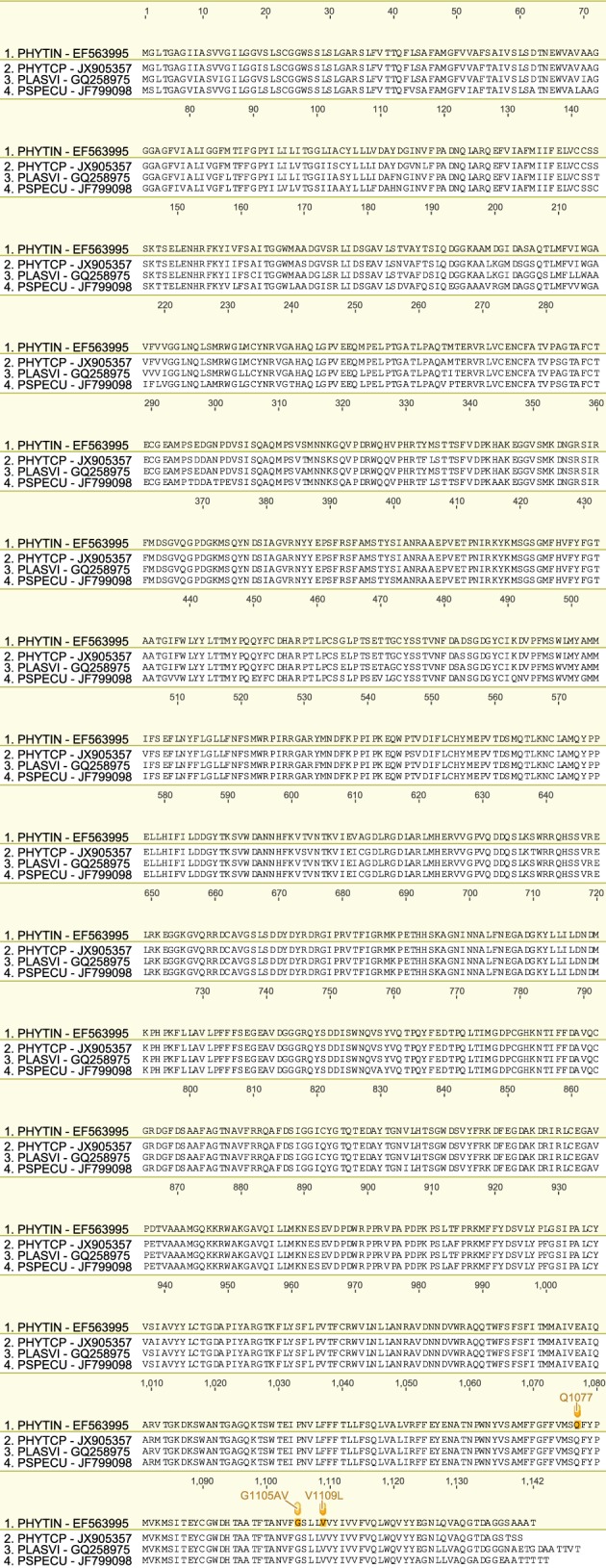
Amino acid sequence alignment of the CesA3 family. Sequences are named by species EPPO code and NCBI gene accession number. Residues highlighted in yellow in the archetype sequence from Phytophthora infestans denote amino acid substitutions associated with fungicide resistance at an orthologous position in any of the sequences. Numerical mutation labels shown above the alignment are based on the position number of the amino acid in the archetype protein.

**Figure 9:**
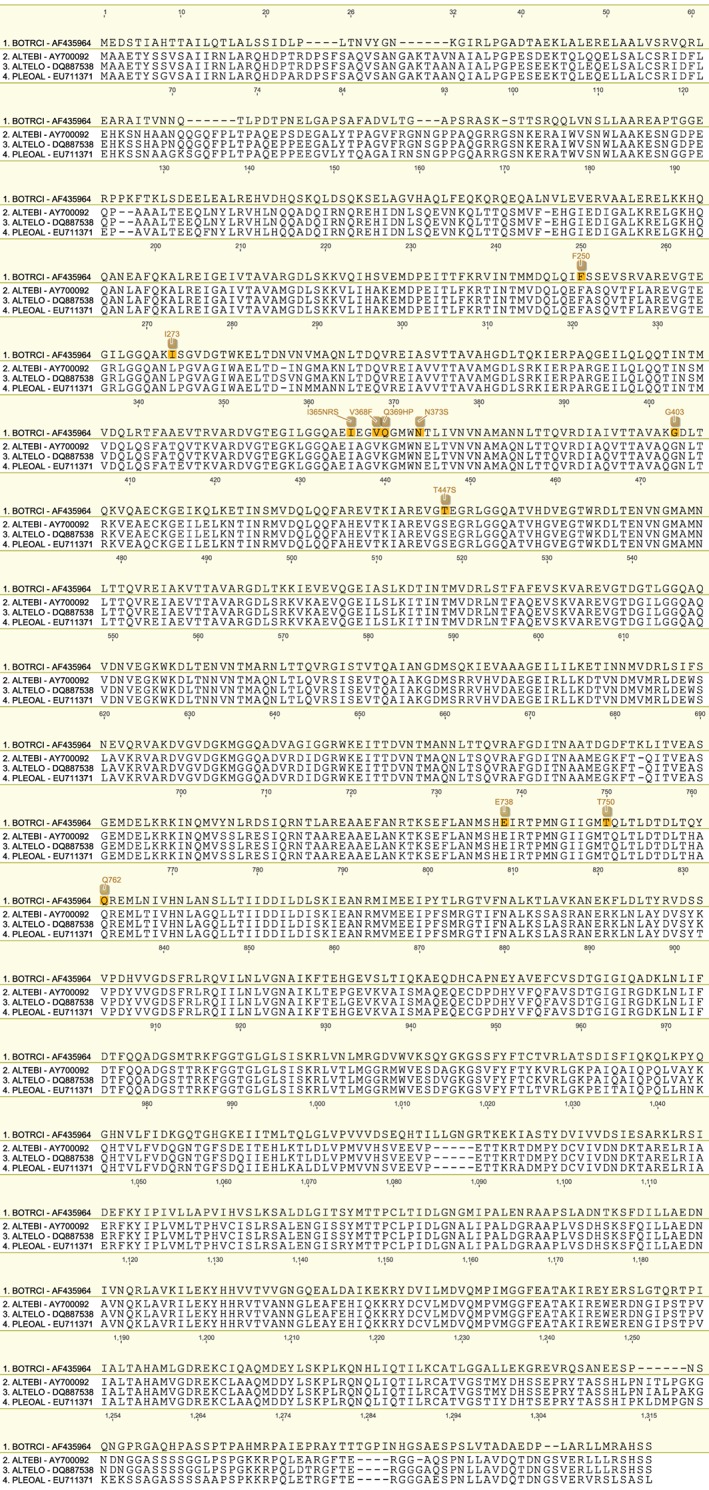
Amino acid sequence alignment of the OS‐1 family. Sequences are named by species EPPO code and NCBI gene accession number. Residues highlighted in yellow in the archetype sequence from Botrytis cinerea denote amino acid substitutions associated with fungicide resistance at an orthologous position in any of the sequences. Numerical mutation labels shown above the alignment are based on the position number of the amino acid in the archetype protein.

We have produced a set of draft alignments of each target protein for which resistance to multiple species has been reported (Figs 1, 2, 3, 4, 5, 6, 7, 8, 9) and tables of putatively orthologous amino acids in other species where fungicide resistance has been reported (Tables 1, 2, 3, 4, 5, 6, 7, 8, 9). The species included in these tables and alignments have been referred to by their European and Mediterranean Plant Protection Organisation (EPPO) codes^9^ as listed in Table 10.

**Table 10:** Abbreviations of species names

Abbreviation (EPPO code)	Name of pathogen
AJELCP	*Ajellomyces capsulatus*
ALTEAL	*Alternaria alternata*
ALTEBI	*Alternaria brassicicola*
ALTELO	*Alternaria longipes*
ALTELY	*Alternaria arborescens*
ALTESO	*Alternaria solani*
ALTETO	*Alternaria tomato*
ASPEFL	*Aspergillus flavus*
ASPEFU	*Aspergillus fumigatus*
ASPEND	*Emericella nidulans*
ASPEPA	*Aspergillus parasiticus*
BOTRCI	*Botryotinia fuckeliana*
CANDAL	*Candida albicans*
COCHHE	*Cochliobolus heterostrophus*
COLLGR	*Glomerella graminicola*
CORYCA	*Corynespora cassiicola*
CERCBE	*Cercospora beticola*
DIDYBR	*Stagonosporopsis cucurbitacearum*
ERYSGH	*Blumeria graminis* f. sp*. hordei*
ERYSGT	*Blumeria graminis* f. sp. *tritici*
EUROOR	*Eurotium oryzae*
FILBNF	*Filobasidiella neoformans*
GIBBFU	*Gibberella fujikuroi*
GIBBZE	*Gibberella zeae*
HELMSO	*Helminthosporium solani*
HYPMOD	*Hypomyces odoratus*
LEPTNO	*Parastagonospora nodorum*
MONGNI	*Monographella nivalis*
MONIFC	*Monilinia fructicola*
MONILA	*Monilinia laxa*
MICDMA	*Microdochium majus*
MYCOFI	*Mycosphaerella fijiensis*
MYCORA	*Didymella rabiei*
NEUSCR	*Neurospora crassa*
PENIAU	*Penicillium aurantiogriseum*
PENIEX	*Penicillium expansum*
PENIIT	*Penicillium italicum*
PHAKPA	*Phakopsora pachyrhizi*
PHYTCP	*Phytophthora capsici*
PHYTDR	*Phytophthora drechsleri*
PHYTIN	*Phytophthora infestans*
PLASVI	*Plasmopara viticola*
PLEOAL	*Pleospora allii*
PODOFU	*Podosphaera fusca*
PODOXA	*Podosphaera xanthii*
PSPECU	*Pseudoperonospora cubensis*
PUCCRT	*Puccinia triticina*
PYRIOR	*Magnaporthe oryzae*
PYRNTE	*Pyrenophora teres*
PYRNTR	*Pyrenophora tritici‐repentis*
PYRPBR	*Pyrenopeziza brassicae*
RHIZSO	*Thanatephorus cucumeris*
RHYNSE	*Rhynchosporium secalis*
SACCCE	*Saccharomyces cerevisiae*
SCLEHO	*Sclerotinia homoeocarpa*
SCLESC	*Sclerotinia sclerotiorum*
SEPTTR	*Zymoseptoria tritici*
UNCINE	*Erysiphe necator*
USTIMA	*Ustilago maydis*
VENTIN	*Venturia inaequalis*

The alignments for *b*‐tubulin and Cytb are essentially colinear in fungi studied to date, and hence there are no changes to be made to the current nomenclature. For the other genes, we have considered four possible methods to generate the alignment. The alignment could be: (1) fitted to the longest gene in the gene set; (2) fitted to a strict consensus alignment; (3) fitted to the gene from the species that is currently the most researched species for the fungicide resistance concerned; (4) fitted to the gene from the species that was the first species for the fungicide resistance concerned.

The aim is to create a set of alignments that would be stable into the foreseeable future and would invoke the least relabelling of mutations that have already been described and published. We favour method 3 (basing the alignment on the species with the most currently described resistance mutations), but also taking into account method 1 (using the longer gene) when alternative species are candidates. We propose that Cyp51A is fitted to ASPEFU (*Aspergillus fumigatus)*, Cyp51B and Cytb are fitted to SEPTRI (*Zymoseptoria tritici)*, *b*‐tubulin to ASPEND (*Aspergillus nidulans)*, the SDH proteins to PYRNTE (*Pyrenophora teres)*, CesA3 to PHYTIN (*Phytophthora infestans)* and Os‐1 to BOTCIN (*Botrytis cinerea)*. For Erg27, mutations associated with resistance have currently been described only in BOTCIN, and thus we propose this species as the archetype. The alignments have been summarised and fungicide resistance associated mutations are given in the tables. By way of example, in Cyp51B the mutation Y136F in ERYSGH would be given the label *Y137F*. In CANDAL (*Candida albicans*) the orthologous amino acid is Y132 and has been mutated to both F and H. The Y132H mutation would therefore be given the label *Y137H*. V151 in SEPTRI is clearly demonstrated to be orthologous to I145F in PHAKPA (*Phakopsora pachyrhizi*). This mutation would be labelled *I151F* in PHAKPA and *V151F* in SEPTRI. The other proposed relabellings are listed in Tables 1, 2, 3, 4, 5, 6, 7, 8, 9.

By examining the species that have amino acid mutations with common labels, we can infer that positions 137, 148, 461, 476, 483 and 524 in Cyp51B are especially important in conferring resistance to triazole fungicides. This is consistent with numerous functional studies.^10^, ^11^ We expect that the alignments should assist the identification of key amino acids in target proteins of newer fungicide classes.

## 4 THE PROPOSAL IN PRACTICE

The system must also allow for mutations to be discovered in new species. The parameters used to make the alignments are described below and can be applied to an alignment between the new species and the archetype. We envisage regularly updating the alignments based on new published knowledge.

A potential problem with the system we propose might occur if an amino acid in a newly described mutant gene corresponded to a gap in the archetype protein's sequence. In such a case, the mutation could be labelled as X50.2Y if it concerned the second extra amino acid after number 50 in the archetype sequence. To our knowledge, no examples of mutations of such poorly conserved amino acids causing resistance have been described, but the possibility remains.

We hope that future studies will refer to the archetype by indicating that the mutation X123Y in the target protein associated with resistance corresponds to the archetype *X145Y* and refer to this paper or a related web page for support.

We suggest that other target genes from medically important fungi (e.g. the FKS1/2 genes that are targets of Echinocandins) and from herbicide‐ and insecticide‐ resistant weeds and insects might also benefit from this approach.

We commend this scheme to the community and seek comment and support. And we urge journal editors to encourage authors to use this new system.

## 5 NOTE ON THE ALIGNMENTS

Amino acid sequences were downloaded from NCBI GenBank and annotated with reported amino acid substitutions^8^, ^12^, ^13^, ^14^, ^15^ using Geneious 6.1.8 software (Biomatters). Alignments of sequences were generated using the ClustalW^16^ algorithm with Blosum scoring matrix, gap opening penalty 10, gap extension penalty 0.5 and free end gaps.

The alignments are available as .doc files and as fasta files in the supporting information.

## Supporting information


**Appendix S1.** Supporting information
