# Supplementary material for: Proposal for a unified nomenclature for target‐site mutations associated with resistance to fungicides
Source: Pest Manag Sci. 2016 Jun 16;72(8):1449–59. doi: 10.1002/ps.4301 (PMC5094580; doi:10.1002/ps.4301)
Supplement: Supplementary file 1 — Appendix S1. Supporting information [file PS-72-1449-s001.docx]

**Cytb Alignment**

....|....| ....|....| ....|....| ....|....| ....|....| ....|....|

5 15 25 35 45 55

SEPTTR_AY247413 MRIWKSHPLF SLVNGYLIDS PQPSNLSYLW NFGSLLGFCL VIQIVTGVTL AMHYNPSVSE

ALTEAL_DQ209283 ---------- ---------- ---------- ---------- ---------- ----------

ALTELY_AY263413 ---------- ---------- ---------- ---------- ---------- ----------

ALTESO_DQ209285 ---------- ---------- ---------- ---------- ---------- ----------

ALTETO_AY263411 ---------- ---------- ---------- ---------- ---------- ----------

BOTRCI_FJ217744 MRIFKSHPLL KLVNSYMIDS PQPSNLSYLW NFGSLLAVCL VIQIITGVTL AMHYNPSVLE

CERCBE_JQ619932 ---------- ---------- ---------- ---------- ---------- ----------

COLLGR_AY285743 ---------- ---------- ---------- ---------- ---------- ----------

ERYSGT_AF343441 ---------- ---------- ---------- ---SLLAVSL VIQIITGVTL AMHYNPSVLE

LEPTNO_ABU49434 MRLLKSHPIL RLANSYLVDS PQPLNLSYMW NFGSLLGVCL IIQIVTGVTL AMHYNPSVAE

MICDMA_FJ560376 ---------- ---------- ---------- ---------- ---------- ----------

MONGNI_FJ560375 ---------- ---------- ---------- ---------- ---------- ----------

MYCOFI_AF343070 ---------- ------IIDX PQPSNISYLW NFGSLLGFCL VIQIVTGVTL AMHYNPSVLE

MYCORA_JQ856020 ---------- ---------- ---------- ---------- ---------- ----------

PHAKPA_DQ209281 ---------- ---------- ---------- ---------- ---------- AMHYIPSVDI

PLASVI_ABF29501 MIRWNKKSLF TVINNHLIDY PTPINLNYFY GFGSLAGIML VVQILTGIFL AMHYTPHIDL

PLEOAL_KJ934233 ---------- ---------- ---------- ---------- ---------- ----------

PODOFU_EF088828 ---------- ---------- ---------- ---------- ---------- ----------

PSPECU_KF679519 MRIFKSHPFL KLVNAYVIDH SQPTNISYMW NFGSLLGVCL VVQIITGVTL AMHYNPSVAE

PYRIOR_AY245425 MRILKSHSLL KLVNSYLIDA SQPSNISYLW NFGSLLAVCL IVQIITGITL AMHYSPSVME

PYRNTE_DQ919067 ---------- ---------- ---------- ---------- ---------- ----------

PYRNTR_DQ919068 ---------- ---------- ---------- ---------- ---------- ----------

RHIZSO_AGK45434 MRLLKSHPIL GLLNSYMVDS PQPANISYMW NFGSLLGMCL VIQILTGAFL AMHYCPSVDM

VENTIN_AF047029 MRILKSHPLL RLANSYIIDS PQPSNISYLW NFGSLLAFCL VIQIITGVTL AMHYNPSVLE

....|....| ....|....| ....|....| ....|....| ....|....| ....|....|

65 75 85 95 105 115

SEPTTR_AY247413 AFNSVEHIMR DVNNGWLIRY LHSNTASAFF FLVYLHVGRG LYYGSYKAPR TLTWTIGTII

ALTEAL_DQ209283 ---------- ---------- ---------- ---------- ---------R TLVWTIGTVI

ALTELY_AY263413 -------IMR DVNNGWLIRY LHSNTASAFF FIVYLHIGRG MYYGSYRAPR TLVWTIGTVI

ALTESO_DQ209285 ---------- ---------- ---------- ---------G LYCGSYRAPR TLVWTIGTVI

ALTETO_AY263411 -------IMR DVNNGWLIRY LHSNTASAFF FIVYLHIGRG MYYGSYRAPR TLVWTIGTVI

BOTRCI_FJ217744 AFNSVEHIMR DVNNGWLIRY LHSNTASAFF FIVYLHIGRG LYYGSYRAPR TLVWTIGVVI

CERCBE_JQ619932 ---------- ---------- -----ASAFF FLVYLHVGRG LYYGSYKAPR TLVWTIGTII

COLLGR_AY285743 ---------- DVNNGWLIRY LHSNTASAFF FLVYLHVGRG MYYGSYRAPR TLVWVIGTII

ERYSGT_AF343441 AFNSVEHIMR DVNNGWLIRY IHSNTASAFF FLVYLHIGRG LYYGSYRAPR TLVWTIGTVI

LEPTNO_ABU49434 AFNSVEHIMR DVNNGWLVRY LHSNTASAFF FIVYLHIGRG LYYGSYRAPR TLVWTIGTVI

MICDMA_FJ560376 ---------- ---------- ---------- ---YLHIGRG MYYGSYRAPR TLVWAIGTVI

MONGNI_FJ560375 ---------- ---------- ---------- ---YLHIGRG MYYGSYRAPR TLVWAIGTVI

MYCOFI_AF343070 AFNSVEHIMR DVNNGWLIRY LHSNTASAFF FLVYLHVGRG LYYGSYKAPR TLVWVIGTII

MYCORA_JQ856020 -------IMR DVNNGWLIRY LHSNTASAFF FIVYLHIGRG MYYGSYRAPR TLVWTIGTVI

PHAKPA_DQ209281 AFISVEHIIR DVEYGWLIRY LHANVASFFF IFVYLHIGRG LYYGSYKSPR TLVWAIGVII

PLASVI_ABF29501 AFNSVEHIMR DVNNGWLIRY IHANGASFFF IVVYIHIFRG LYYGSYITPR EALWCSGVII

PLEOAL_KJ934233 ---------- ---------- ---------- ---------- ---------- -LVWTIGTVI

PODOFU_EF088828 ---------- DVNNGWLVRY LHANTASAFF FIMYLHIGRG LYYGSYKSPR TLTWAIGTVI

PSPECU_KF679519 AFNSIEHIMR DVNNGWLVRY LHSNTASAFF FLVYLHIGRG IYYGSYRAPR TLAWVIGTII

PYRIOR_AY245425 AFNSIEHIMR DVNNGWLVRY LHSNTASAFF FLVYLHIGRG IYYGSYRAPR TLVWAIGTVI

PYRNTE_DQ919067 -FNSVEHIMR DVNNGWLIRY LHSNTASAFF FVVYLHIGRG MYYGSYRAPR TLVWTIGTVI

PYRNTR_DQ919068 ---------- ---------- ---------- ---------- ---------- ----------

RHIZSO_AGK45434 VFISVEHIMR DVNYGWAVRY THANTASFFF IFVYFHVARG LYYGSYRSPR VLLWSIGVVM

VENTIN_AF047029 AFNSVEHIMR DVNNGWLIRY LHANTASAFF FIVYLHMGRG LYYGSYRAPR TLVWTLGVII

**Cytb Alignment (continued)**

....|....| ....|....| ....|....| ....|....| ....|....| ....|....|

125 135 145 155 165 175

SEPTTR_AY247413 LVLMMATAFL GYVLPYGQMS LWGATVITNL LSAIPWVGQD IVEFVWG-GF --------SV

ALTEAL_DQ209283 FILMMATAFL GYVLPYGQMS LWGATVITNL MSAIPWVGQD IVEFIWG-GF --------SV

ALTELY_AY263413 FILMMATAFL GYVLPYGQMS LWGATVITNL MSAIPWIGQD IV-------- ----------

ALTESO_DQ209285 FILMMATAFL GYVLPYGQMS LWGATVITNL MSAIPWVGQD IVEFIWG-GF --------SV

ALTETO_AY263411 FILMMATAFL GYVLPYGQMS LWGATVITNL MSAIPWIGQD IV-------- ----------

BOTRCI_FJ217744 FILMIVTAFL GYVLPYGQMS LWGATVITNL MSAVPWIGQD IVEFLWG-GF --------SV

CERCBE_JQ619932 LVLMMATAFL GYVLPYGQMS LWGATVITNL MSAIPWVGQD IVEFLWG-GF --------SV

COLLGR_AY285743 LVAMMGIGFL GHVLPYGQMS LWGATVITNL ISAIPWIGQD IVEFVWG-GF --------SV

ERYSGT_AF343441 FILMIVTAFL GYVLPYGQMS HWGATVITNL MSAIPWIGQD IVEFLWG-GF --------SV

LEPTNO_ABU49434 FILMMATAFL GYVLPYGQMS LWGATVITNL MSAIPWVGQD IVEFIWGVCF KWSYQHKCSV

MICDMA_FJ560376 LILMDGTAFL GYVLPYGQMS LWGATVITNL ISAIPWIGQD IVE------- ----------

MONGNI_FJ560375 LILMDGTAFL GYVLPYGQMS LWGATVITNL ISAIPWIGQD IVE------- ----------

MYCOFI_AF343070 LVLMMATAFL GYVLPYGQMS LWGATVITNL MSAIPWVGQD IVEFIWG-GL --------SV

MYCORA_JQ856020 FILMMATAFL GYVLPYGQMS LWAATVITNL MSAIPWVGQD IVEFIWG-GF --------SV

PHAKPA_DQ209281 LIVIIATAFI GYVLPYGQIS LWGATVITNL ISAIPWIGGD LVEFIWG-GF --------SV

PLASVI_ABF29501 FILMMATAFM GYVLPWGQMS FWGATVITNL FSAIPLIGKE VVDWLWG-GF --------AV

PLEOAL_KJ934233 FILMMATAFL GYVLPYGQMS LWGATVITNL MSAIPWVGQD IVEFIWG-GF --------SV

PODOFU_EF088828 VILMMATAFL GYVLPYGQMS LWGATVITNL MSAIPWIGQD IVEFIWG-GF --------SV

PSPECU_KF679519 FIVMVVTAFL GYVLPYGQMS LWAATVITNL ISAVPWIGQD IVEFIWG-GF --------SV

PYRIOR_AY245425 LILMMAIGFL GYVLPYGQMS LWGATVITNL ISAIPWIGQD IVEFIWG-GF --------SV

PYRNTE_DQ919067 FILMMATAFL GYVLPYGQMS LWG------- ---------- ---------- ----------

PYRNTR_DQ919068 -------AFL GYVLPYGQMS LWGATVITNL MSAIPWV--- ---------- ----------

RHIZSO_AGK45434 LVMMMGIAFL GYVLPYGQMS LWGATVITNL LSAIPWIGQD FVQLVWG-GF --------SV

VENTIN_AF047029 FILMIVTAFL GYVLPYGQMS LWGATVITNL MSAIPWIGQD IVEFLWG-GF --------SV

....|....| ....|....| ....|....| ....|....| ....|....| ....|....|

185 195 205 215 225 235

SEPTTR_AY247413 NNATLNRFFA LHFVLPFVLA ALVLMHLIAL HDTAGSGNPL GVSGNYDRLP FAPYFIFKDL

ALTEAL_DQ209283 NNATLNRFFS LHFVLPFVLA ALALMHLIVL HDTAGSGNPL GVSGNYERIS FAPYFIFKDL

ALTELY_AY263413 ---------- ---------- ---------- ---------- ---------- ----------

ALTESO_DQ209285 NNATLNRFFS LHFVLPFVLA ALALMHLIVL HDTAGSGNPL GVSGNYERIS FAPYFIFKDL

ALTETO_AY263411 ---------- ---------- ---------- ---------- ---------- ----------

BOTRCI_FJ217744 NNATLNRFFA LHFVLPFVLA ALALMHLIAL HDSAGSGNPL GISGNYDRLA FAPYFLFKDL

CERCBE_JQ619932 NNATLNRFFA LHFVLPFVLA ALALMHLIAL HDSAGSGNPL GVSGNYDRLP FAPYFIFKDL

COLLGR_AY285743 NNATLNRFFA LHFVLPFVLA ALVLMHLIAL HDTVGSSNPL GVSGNYDRIP FAPYYLFKDL

ERYSGT_AF343441 NNATLNRFFA LHFVLPFVLA ALALMHLIAL HDSAGSGNPL GVSGNYDRLP MAPYFLFKDL

LEPTNO_ABU49434 NNATLNRFFS LHFVLPFVLA ALVLMHLIVL HDTSGSGNPL GISGNYERIP FAPYFIFKDL

MICDMA_FJ560376 ---------- ---------- ---------- ---------- ---------- ----------

MONGNI_FJ560375 ---------- ---------- ---------- ---------- ---------- ----------

MYCOFI_AF343070 NNATLNRFFA LHFVFPFVLA ALALMHLIAL HDSAGSGNPL GVSGNYDRLP FAPYFIFKDL

MYCORA_JQ856020 NNATLNRFFS LHFVLPFVLA ALALMHLIVL HDTAGSGNPL GVSGNYERIS FAPYFIFKDL

PHAKPA_DQ209281 SNATLNRFFS IHFVLPFIIA ALAAIHLLTL HEH-GSSNPL GVTGNADRLP IAPYFIFKDL

PLASVI_ABF29501 DNPTLNRFFS LHFTFPFVIV GAVLIHLILL HEV-GSNNPL GITLKTENIP FYPYFYTKDL

PLEOAL_KJ934233 NNATLNRFFS LHFVLPFVLA ALALMHLIVL HDTAGSGNPL GVSGNYDRMP FAPYLIFKDL

PODOFU_EF088828 NNATLNRFFA LHFLLPFVLA ALVVMHLIAY HDVVGSGNPL GISGNYDRLP FAPYFVFKDL

PSPECU_KF679519 NNATLNRFFA LHFVLPFVLA ALVIMHLIAV HETAGASNPL GVPGYYDRVP MAPYFLFKDL

PYRIOR_AY245425 NNATLNRFFA LHFVLPFVLA ALVLMHLIAL HDTAGSSNPL GVSGNYDRIT FAPYYLFKDL

PYRNTE_DQ919067 ---------- ---------- ---------- ---------- ---------- ----------

PYRNTR_DQ919068 ---------- ---------- ---------- ---------- ---------- ----------

RHIZSO_AGK45434 SNATLNRFFS LHYLLPFVLA ALVAMHFIAL HEH-GSSNPL GISGNVDRLS FHPFFTFKDL

VENTIN_AF047029 NNATLNRFFA LHFVLPFVLA ALALMHLIAL HDSAGSGNPL GVSGNFDRLP FAPYFIFKDL

**Cytb Alignment (continued)**

....|....| ....|....| ....|....| ....|....| ....|....| ....|....|

245 255 265 275 285 295

SEPTTR_AY247413 ITIFLFIIVL SIFIFFMPNV LGDSENYVMA NPMQTPPAIV PEWYLLPFYA ILRSIPNKLL

ALTEAL_DQ209283 ITIFAFIFVL SLFVFFMPNV LGDSENYVVA NPMQTPAAIV PEWYLLPFYA ILRSIP----

ALTELY_AY263413 ---------- ---------- ---------- ---------- ---------- ----------

ALTESO_DQ209285 ITIFAFIFVL SLFVFFMPNV LGDSENYVVA NPMQTPAAIV PEWYLLPFYA ILRSIPNKLL

ALTETO_AY263411 ---------- ---------- ---------- ---------- ---------- ----------

BOTRCI_FJ217744 ITIFLFIIIL SIFVFFMPNV LGDSDNYIMA NPMQTPPAIV PEWYLLPFYA ILRSIPNKLL

CERCBE_JQ619932 ITIFLFIIVL SVFVFFMPNV LGDSENYVVA NPMQTPPAIV PEWYLLPFYA ILRSIPNKLL

COLLGR_AY285743 ITIFMFVFGL SLFVFFMPNV LGDSDNYIMA NPMQTPAAIV PEWYLLPFYA I---------

ERYSGT_AF343441 ITIFLFIIIL SMFVFFMSNV LGDSENYVMA NPMQTPAAIV PEWYLLPFYA ILRSIPNKLL

LEPTNO_ABU49434 ITIFAFIFVL SLFVFFMPNV LGDSENYVVA NPMQTPAAIV PEWYLLPFYA ILRSIPNKLL

MICDMA_FJ560376 ---------- ---------- ---------- ---------- ---------- ----------

MONGNI_FJ560375 ---------- ---------- ---------- ---------- ---------- ----------

MYCOFI_AF343070 ITIFLLIVVL SIFVFFMPNV LGDSENYVMA NPMQTPPAIV PEWYLLPFYA ILRSIPNKLL

MYCORA_JQ856020 ITVFAFIFVL SLFVFFMPNV LGDSENYVMA NPMQTPAAIV PEWYLLPFYA ILRSIPNKLL

PHAKPA_DQ209281 VTIFIFFLVL AIFVMYAPNL IGHSDNYIPA NPIQTPASIV PEWYLLPFYA ILRA------

PLASVI_ABF29501 FGLIVLFLIF FIFIFYYPNT LGHPDNYIEA NPMKTPLHIV PEWYFLPFYA ILRSIPNKIG

PLEOAL_KJ934233 ITIFAFIFVL SLFVFFMPNV LGD------- ---------- ---------- ----------

PODOFU_EF088828 ITIFIFFIVL SVFVFFMPNA LGDSDNYIMA NPMQTPPAIV PEWYLLPFYA ILRSIPNKLL

PSPECU_KF679519 ITIFVFFFGL SIFVFYMPNV LGDSDNYIMA NPMQTPPAIV PEWYLLPFYA ILRSIPNKLL

PYRIOR_AY245425 ITIFIFIFVL SAFVFFMPNV LGDSDNYIMA NPMQTPAAIV PEWYLLPFYA ILRSIPNKLL

PYRNTE_DQ919067 ---------- ---------- ---------- ---------- ---------- ----------

PYRNTR_DQ919068 ---------- ---------- ---------- ---------- ---------- ----------

RHIZSO_AGK45434 VTVFLFLLTL SIFVFYMPNA LGHSDNYIPA NPMSTPASIV PEWDLLPYYA ILRSIPNKLV

VENTIN_AF047029 ITIFLFILGL SIFVFFAPNI LGDSENYVVA NPMQTPPAIV PEWYLLPFYA ILRSIPNKLL

....|....| ....|....| ....|....| ....|....| ....|....| ....|....|

305 315 325 335 345 355

SEPTTR_AY247413 GVIAMFSAIL IIMIMPITDL GRSRGLQFRP LSKITFYIFV ANFLVLMQLG ANHVESPFIE

ALTEAL_DQ209283 ---------- ---------- ---------- ---------- ---------- ----------

ALTELY_AY263413 ---------- ---------- ---------- ---------- ---------- ----------

ALTESO_DQ209285 GVIA------ ---------- ---------- ---------- ---------- ----------

ALTETO_AY263411 ---------- ---------- ---------- ---------- ---------- ----------

BOTRCI_FJ217744 GVIAMLSAIL ILLAMPFTDL SRSRGIQFRP LSKIAFYIFI ANFLILMVLG AKHVESPYIE

CERCBE_JQ619932 GVIAMFSA-- ---------- ---------- ---------- ---------- ----------

COLLGR_AY285743 ---------- ---------- ---------- ---------- ---------- ----------

ERYSGT_AF343441 GVIAMFSAIL ALLAMPFTDL SRSRGLQFKP LNKAAFYIFI GNFVILMVLG AKHVESPYIE

LEPTNO_ABU49434 GVIAMFSAIL ILLLLPITDV SRSRGMQFRP LSKWAFFVFV ANFLILMQLG AKHVESPFIE

MICDMA_FJ560376 ---------- ---------- ---------- ---------- ---------- ----------

MONGNI_FJ560375 ---------- ---------- ---------- ---------- ---------- ----------

MYCOFI_AF343070 GVIAMFSAIL IIMIMPFTDL GRSRGLQFRP LSKIAFFVFV ANFLVLMQXG AKHVESPYIE

MYCORA_JQ856020 G--------- ---------- ---------- ---------- ---------- ----------

PHAKPA_DQ209281 ---------- ---------- ---------- ---------- ---------- ----------

PLASVI_ABF29501 GVIAMFGSLI ILLTIPFTNS SEIRSTTFRP IFKVCYWLLV VAFILLGWVG QCPVEYPYTE

PLEOAL_KJ934233 ---------- ---------- ---------- ---------- ---------- ----------

PODOFU_EF088828 GV-------- ---------- ---------- ---------- ---------- ----------

PSPECU_KF679519 GVIAMLAAIL IILALPLVDL GRTQGLQFRP LSKVVFWIFV VNFLILMQLG AKHVESPFIE

PYRIOR_AY245425 GVIAMFSAIL AIMLLPVTDL GRSRGLQFRP FSKIAFWVFV ANFLVLMQLG AKHVEDPFIL

PYRNTE_DQ919067 ---------- ---------- ---------- ---------- ---------- ----------

PYRNTR_DQ919068 ---------- ---------- ---------- ---------- ---------- ----------

RHIZSO_AGK45434 GVLAMFASLL ILLIMPIVDT SRIRGNQFRP LMKFFFWVFV ANFFILMWIG SQHPNSPFVE

VENTIN_AF047029 GVIAMFAAIV ILLVMPFTDL GRSRGIQFRP LSKIAYYFFI ANFLILMKLG AKHVESPFIE

**Cytb Alignment (continued)**

....|....| ....|....| ....|....| ....|....| ....|...

365 375 385 395 405

SEPTTR_AY247413 FGQISTVLYF SHFLIIVPLV SLIENTLVDM HLNNTIT--- --------

ALTEAL_DQ209283 ---------- ---------- ---------- ---------- --------

ALTELY_AY263413 ---------- ---------- ---------- ---------- --------

ALTESO_DQ209285 ---------- ---------- ---------- ---------- --------

ALTETO_AY263411 ---------- ---------- ---------- ---------- --------

BOTRCI_FJ217744 FGQISTVIYF AHFLIIVPFI SLLENSLVEL AVLTKEKPSR --------

CERCBE_JQ619932 ---------- ---------- ---------- ---------- --------

COLLGR_AY285743 ---------- ---------- ---------- ---------- --------

ERYSGT_AF343441 LGQFSTLLYF SHFLLIVPLV NFLENTLIVL SCRSS----- --------

LEPTNO_ABU49434 FGQISTVLYF LYFTVVMYGV TFIENTFVDL NFYTNTKHSS RFNFVTKK

MICDMA_FJ560376 ---------- ---------- ---------- ---------- --------

MONGNI_FJ560375 ---------- ---------- ---------- ---------- --------

MYCOFI_AF343070 FGQIRTVLYF SHFLIIVPLL SILENTLIDL HLNNPK---- --------

MYCORA_JQ856020 ---------- ---------- ---------- ---------- --------

PHAKPA_DQ209281 ---------- ---------- ---------- ---------- --------

PLASVI_ABF29501 IGIISMIYYF SFFLIIIPFL GKIETYLIRY KCNK------ --------

PLEOAL_KJ934233 ---------- ---------- ---------- ---------- --------

PODOFU_EF088828 ---------- ---------- ---------- ---------- --------

PSPECU_KF679519 FGQISTVLYF SHFLIIIPLT SLIENVLISV NAR---FQQA Q-------

PYRIOR_AY245425 LGQLSTVLYF SYFVAILPLA SYLDNSLTDL SNKSELFLNK TN------

PYRNTE_DQ919067 ---------- ---------- ---------- ---------- --------

PYRNTR_DQ919068 ---------- ---------- ---------- ---------- --------

RHIZSO_AGK45434 VGQVATALYF AWFVIIVPLV GIVENTLMDL ALT----SHD S-------

VENTIN_AF047029 FGQISTVLYF SHFVIIVPLV SLIENTLVDL HLHNTLSLKN VF------

**Beta-Tubulin Alignment**

....|....| ....|....| ....|....| ....|....| ....|....| ....|....|

5 15 25 35 45 55

ASPEND__M17519 MREIVHLQTG QCGNQIGAAF WQTISGEHGL DGSGVYNGTS DLQLERMNVY FNEASGNKYV

BOTRCI_Z69263 MREIVHLQTG QCGNQIGAAF WQTISGEHGL DGSGVYNGTS DLQLERMNVY FNEASGNKYV

CERCBE_AY856373 MREIVHLQTG QCGNQIGAAF WQTISGEHGL DGSGVYNGTS DLQLERMNVY FNEASGNKYV

COCHHE_AB009971 MREIVHLQTG QCGNQIGAAF WQTISGEHGL DGSGVYNGTS DLQLERMNVY FNEASNNKFV

GIBBFU_U27303 MREIVHLQTG QCGNQIGAAF WQTISGEHGL DSNGVYNGTS ELQLERMSVN FNERSGNKYV

GIBBFU_KF771181 MREIVHVQVG QCGNQVGSSF WQTVSGEHGV DGSGSYNGTD DQQRERIDVY FAEATKDKYV

GIBBZE_FJ214663 MREIVHIQVG QCGNQVGSSF WSTVSKEHGI DGSGAYHGTS DQQRERINVY FAEGGNDKYV

HELMSO_Y10670 ---------- ---------- ---------- ---------- --QLERMNVY FNEASGNKFV

HYPMOD_Y12256 MREIVHLQTG QCGNQVGAAF WQTISGEHGL DSSGIYNGSS ELQLERMNVY FNEASDNKYV

LEPTNO_S56922 MREIVYLQTG QCGNQIGAAF WQTISGEHGL DGSGVYNGTS DLQLERMNVY FNEASGNKFV

MONIFC_AY283676 MREIVHLQTG QCGNQIGAAF WQTISGEHGL DGSGVYNGTS DLQLERMNVY FNEASGNKYV

MONILA_AY349149 MREIVHLQTG QCGNQIGAAF WQTISGEHGL DGSGVYNGTS DLQLERMNVY FNEASGNKYV

NEUSCR_M13630 MREIVHLQTG QCGNQIGAAF WQTISGEHGL DASGVYNGTS ELQLERMNVY FNEASGNKYV

PENIAU_JN112033 ---------- ---------- ---------- ---------- ---------- ----------

PENIEX_KGO42666 MREIVHLQTG QCGNQIGAAF WQTISGEHGL DGDGQYNGTS DLQLERMNVY FNHASGDKYV

PENIIT_KGO72779 MREIVHLQTG QCGNQIGAAF WQTISGEHGL DGDGQYNGTS DLQLERMNVY FNHASGDKYV

PYRPBR_KC342227 MREIVHLQTG QCGNQIGAAF WQTISGEHGL DGSGVYNGTS DLQLERMNVY FNEASGNKYV

RHYNSE_X81046 MREIVHLQTG QCGNQIGAAF WQTISGEHGL DGSGVYNGTS DLQLERLNVY FNEASGNKYV

SCLEHO_KF765483 MREIVHLQTG QCGNQIGAAF WQTISGEHGL DGSGVYNGTS DLQLERMNVY FNEASGNKYV

SCLESC_XM_001594794 MREIVHLQTG QCGNQIGAAF WQTISGEHGL DGSGVYNGTS DLQLERMNVY FNEASGNKYV

VENTIN_M97951 MREIVHLQTG QCGNQIGAAF WQTISGEHGL DGSGVYNGTS DLQLERMNVY FNEASGNKFV

....|....| ....|....| ....|....| ....|....| ....|....| ....|....|

65 75 85 95 105 115

ASPEND__M17519 PRAVLVDLEP GTMDCVRAGP FGELFRPDNF VFGQSGAGNN WAKGHYTEGA ELVDNVVDVV

BOTRCI_Z69263 PRAVLVDLEP GTMDAVRAGP FGQLFRPDNF VFGQSGAGNN WAKGHYTEGA ELVDQVLDVV

CERCBE_AY856373 PRAVLVDLEP GTMDAVRAGP XGQLFRPDNF VFGQSGAGNN WAKGHYTEGA ELVDQVLDVV

COCHHE_AB009971 PRAVLVDLEP GTMDAVRAGP FGQLFRPDNF VFGQSGAGNN WAKGHYTEGA ELVDQVLDVV

GIBBFU_U27303 PRAVLVDLEP GTMDAVRAGP FGQLFRPDNF VFGQSGAGNN WAKGHYTEGA ELVDQVLDVV

GIBBFU_KF771181 PRAVLVDLEP GPQDAIRAGP LGQLFRPDNF VAGNASAGNN WAKGHYTEGA ELVEEAIDVV

GIBBZE_FJ214663 PRAVLVDLES GPQDAIRAGP LGQLFRPDNF VAGEASAGNN WAKGHYTEGA ELVEEAIDVV

HELMSO_Y10670 PRAVLVDLEP GTMDAVRAGP FGQLFRPDNF VFGQSGAGNN WAKGHYTEGA ELVDQVLDVV

HYPMOD_Y12256 PRAVLVDLEP GTIDAVKSGP FGQLFRPDNF IFGQSSAGNN WAKGHYTEGA ELVDAVLDVV

LEPTNO_S56922 PRAVLVDLEP GTMDAVRAGP FGQLFRPDNF VFGQSGAGNN WAKGHYTEGA ELVDQVLDVV

MONIFC_AY283676 PRAVLVDLEP GTMDAVRAGP FGQLFRPDNF VFGQSGAGNN WAKGHYTEGA ELVDQVLDVV

MONILA_AY349149 PRAVLVDLEP GTMDAVRAGP FGQLFRPDNF VFGQSGAGNN WAKGHYTEGA ELVDQVLDVV

NEUSCR_M13630 PRAVLVDLEP GTMDAVRAGP FGQLFRPDNF VFGQSGAGNN WAKGHYTEGA ELVDQVLDVV

PENIAU_JN112033 PRAVLVDLEP GTMDAVRSGP FGKLFRPDNF VFGQSGAGNN WAKGHYTEGA ELVDQVLDVV

PENIEX_KGO42666 PRAVLVDLEP GTMDAVRSGP FGKLFRPDNF VFGQSGAGNN WAKGHYTEGA ELVDQVLDVV

PENIIT_KGO72779 PRAVLVDLEP GTMDAVRSGP FGKLFRPDNF VFGQSGAGNN WAKGHYTEGA ELVDQVLDVV

PYRPBR_KC342227 PRAVLVDLEP GTMDAVRAGP FGQLFRPDNF VFGQSGAGNN WAKGHYTEGA ELVDQVLDVV

RHYNSE_X81046 PRAVLVDLEP GTMDAVRAGP FGQLFRPDNF VFGQSGAGNN WAKGHYTEGA ELVDQVLDVV

SCLEHO_KF765483 PRAVLVDLEP GTMDAVRAGP FGQLFRPDNF VFGQSGAGNN WAKGHYTEGA ELVDQVLDVV

SCLESC_XM_001594794 PRAVLVDLEP GTMDAVRAGP FGQLFRPDNF VFGQSGAGNN WAKGHYTEGA ELVDQVLDVV

VENTIN_M97951 PRAVLVDLEP GTMDAVRAGP FGQLFRPDNF VFGQSGAGNN WAKGHYTEGA ELVDQVLDVV

....|....| ....|....| ....|....| ....|....| ....|....| ....|....|

125 135 145 155 165 175

ASPEND__M17519 RREAEGCDCL QGFQITHSLG GGTGAGMGTL LISKIREEFP DRMMATFSVV PSPKVSDTVV

BOTRCI_Z69263 RREAEGCDCL QGFQITHSLG GGTGAGMGTL LISKIREEFP DRMMATFSVV PSPKVSDTVV

CERCBE_AY856373 RREAEGCDCL QGFQITHSLG GGTGAGMGTL LISKIREEFP DRMMATFSVM PSPKVSDTVV

COCHHE_AB009971 RREAEGCDCL QGFQITHSLG GGTGAGMGTL LISKIREEFP DRMMATYSVV PSPKVSDTVV

GIBBFU_U27303 RREAEGCDCL QGFQITHSLG GGTGAGMGTL LISKIREEFP DRMMATFSVV PSPKVSDTVV

GIBBFU_KF771181 RHEVENCEHL QGFQLTHSLG GGTGSGMGTL LLSKIREEFP DRMMATFSVM PSPKVSDTVV

GIBBZE_FJ214663 RREVENCDHL QGFQLTHSLG GGTGSGMGTL LLSKIREEFP DRMMATFSVM PSPKVSDTVV

HELMSO_Y10670 RREAEGCDCL QGFQITHSLG GGTGAGMGTL LISKIREEFP DRMMATFSVM PSPKVSDTVV

HYPMOD_Y12256 RREAEGCDCL QGFQITHSLG GGTGSGMGTL LISKIREEFP DRMMATFSVV PSPKVSDTVV

LEPTNO_S56922 RREREGCDCL QGFQITHSLG GGTGAGMGTL LISKIREEFP DRMMATFSVV PSPKVSDTVV

MONIFC_AY283676 RREAEGCDCL QGFQITHSLG GGTGAGMGTL LISKIREEFP DRMMATFSVV PSPKVSDTVV

MONILA_AY349149 RREAEGCDCL QGFQITHSLG GGTGAGMGTL LISKIREEFP DRMMATFSVV PSPKVSDTVV

NEUSCR_M13630 RREAEGCDCL QGFQITHSLG GGTGAGMGTL LISKIREEFP DRMMATYSVV PSPKVSDTVV

PENIAU_JN112033 RREAEGCDCL QGFQITHSLG GGTGAGMGTL LISKIREEFP DRMMATFSVV PSPKVSDTVV

PENIEX_KGO42666 RREAEGCDCL QGFQITHSLG GGTGAGMGTL LISKIREEFP DRMMATFSVV PSPKVSDTVV

PENIIT_KGO72779 RREAEGCDCL QGFQITHSLG GGTGAGMGTL LISKIREEFP DRMMATFSVV PSPKVSDTVV

PYRPBR_KC342227 RREAEGCDCL QGFQITHSLG GGTGAGMGTL LISKIREEFP DRMMATFSVV PSPKVSDTVV

RHYNSE_X81046 RREAEGCDCL QGFQITHSLG GGTGAGMGTL LISKIREEFP DRMMATFSVV PSPKVSDTVV

SCLEHO_KF765483 RREAEGCDCL QGFQITHSLG GGTGAGMGTL LISKIREEFP DRMMATFSVV PSPKVSDTVV

SCLESC_XM_001594794 RREAEGCDCL QGFQITHSLG GGTGAGMGTL LISKIREEFP DRMMATFSVV PSPKVSDTVV

VENTIN_M97951 RREAEGCDCL QGFQITHSLG GGTGAGMGTL LISKIREEFP DRMMATFSVV PSPKVSDTVV

**Beta-Tubulin Alignment (continued)**

....|....| ....|....| ....|....| ....|....| ....|....| ....|....|

185 195 205 215 225 235

ASPEND__M17519 EPYNATLSVH QLVEHSDETF CIDNEALYDI CMRTLKLSNP SYGDLNHLVS AVMSGVTTCL

BOTRCI_Z69263 EPYNATLSVH QLVENSDETF CIDNEALYDI CMRTLKLSNP SYGDLNHLVS AVMSGVTTCL

CERCBE_AY856373 EPYNATLSVH QLVENSDETF CIDNEALYDI CMRTLKLNNP SYGDLNHLVS AVMSGVTTCL

COCHHE_AB009971 EPYNATLSIH QLVENSDETF CIDNEALYDI CMRTLKLNNP SYGDLNHLVS AVMSGVTTCL

GIBBFU_U27303 EPYNATLSVH QLVENSDETF CIDNEALYDI CMRTLKLSNP SYGDLNYLVS AVMSGVTTCL

GIBBFU_KF771181 EPYNATLSLN QLVENSDETF CIDNEALYDI YEKTLKIADP SYADLNYLIS TVMAGVTTCF

GIBBZE_FJ214663 EPYNATLSLN QLVENSDETF CIDNEALYDI YERTLKIADP SYADLNYLIS TVMAGVTTCF

HELMSO_Y10670 EPYNATLSVH QLVENSDETF CIDNEALYDI CMRTLKLNNP SYGDLNHLVS AVMSGVTTCL

HYPMOD_Y12256 EPYNATLSIH QLVENSDETF CIDNEALYDI CMKTLKLPNP AYADLNYLVS AVMSGVTTCL

LEPTNO_S56922 EPYNATLSIH QLVENSDETF CIDNEALYDI CMRTLKLNNP SYGDLNHLVS AVMSGVTTCL

MONIFC_AY283676 EPYNATLSVH QLVENSDETF CIDNEALYDI CMRTLKLSNP SYGDLNHLVS AVMSGVTTCL

MONILA_AY349149 EPYNATLSVH QLVENSDETF CIDNEALYDI CMRTLELSNP SYGDLNHLVS AVMSGVTTCL

NEUSCR_M13630 EPYNATLSVH QLVENSDETF CIDNEALYDI CMRTLKLSNP SYGDLNHLVS AVMSGVTVSL

PENIAU_JN112033 EPYNATLSVH QLVEHSDETF CIDNEALYDI CMRTLKLSQP SYGDLNHLVS AVMSGVTTSL

PENIEX_KGO42666 EPYNATLSVH QLVEHSDETF CIDNEALYDI CMRTLKLSQP SYGDLNHLVS AVMSGVTTSL

PENIIT_KGO72779 EPYNATLSVH QLVEHSDETF CIDNEALYDI CMRTLKLSQP SYGDLNHLVS AVMSGVTTSL

PYRPBR_KC342227 EPYNATLSIH QLVENSDETF CIDNEALYDI CMRTLKLTNP SYGDLNHLVS AVMSGVTTCL

RHYNSE_X81046 EPYNATLSIH QLVENSDETF CIDNEALYDI CMRTLKLTNP SYGDLNHLVS AVMSGVTTCL

SCLEHO_KF765483 EPYNATLSVH QLVENSDETF CIDNEALYDI CMRTLKLSNP SYGDLNHLVS AVMSGVTTCL

SCLESC_XM_001594794 EPYNATLSVH QLVENSDETF CIDNEALYDI CMRTLKLSHP SYGDLNHLVS AVMSGVTTCL

VENTIN_M97951 EPYNATLSVH QLVENSDETF CIDNEALYDI CMRTLKLNNP SYGDLNHLVS AVMSGVTTCL

....|....| ....|....| ....|....| ....|....| ....|....| ....|....|

245 255 265 275 285 295

ASPEND__M17519 RFPGQLNSDL RKWAVNMVPF PRLHFFMVGF APLTSRGAYS FRAVSVPELT QQMFDPKNMM

BOTRCI_Z69263 RFPGQLNSDL RKLAVNMVPF PRLHFFMVGF APLTSRGAHS FRAVTVPELT QQMYDPKNMM

CERCBE_AY856373 RFPGQLNSDL RKLAVNMVPF PRLHFFMVGF APLTSRGAHS FRAVTVPELT QQIFDPKNMM

COCHHE_AB009971 RFPGQLNSDL RKLAVNMVPF PRLHFFMVGF APLTSRGAHS FRAVTVPELT QQMFDPKNMM

GIBBFU_U27303 RFPGQLNSDL RKLAVNMVPF PRLHFFMVGF APLTSRGAHS FRAVSVPELT QQMFDPKNMM

GIBBFU_KF771181 RFPGQLNSDL RKLAVNMIPF PRLHFFMVGF APLTGRKMQG FQHLTVPSLA QQIFDNKNVM

GIBBZE_FJ214663 RFPGQLNSDL RKLAVNMIPF PRLHFFMVGF APLTGRNMKT FQHVTVPGLA QQIFDNKNIM

HELMSO_Y10670 RFPGQLNSDL RKLAVNMVPF PRLHFFMVGF APLTSRGAHS FRAVSVPELT QQMFDPKNMM

HYPMOD_Y12256 RFPGQLNSDL RKLAVNMVPF PRLHFFMVGF APLTSRGAHS FRAVTVPELT QQMFDPKNMM

LEPTNO_S56922 RFPGQLNSDL RKLAVNMVPF PRLHFFMVGF APLTSRGAHS FRAVTVPELT QQMFDPKNMM

MONIFC_AY283676 RFPGQLNSDL RKLAVNMVPF PRLHFFMVGF APLTSRGAHS FRAVTVPELT QQMYDPKNMM

MONILA_AY349149 RFPGQLNSDL RKLAVNMVPF PRLHFFMVGF APLTSRGAHS FRAVTVPELT QQMYDPKNMM

NEUSCR_M13630 RFPGQLNSDL RKLAVNMVPF PRLHFFMVGF APLTSRGAHH FRAVSVPELT QQMFDPKNMM

PENIAU_JN112033 RFPGQLNSDL RKLAVNMVPF PRLHFFMVGF APLTSRGGQS YRQVSVPELT QQMFDPKNMM

PENIEX_KGO42666 RFPGQLNSDL RKLAVNMVPF PRLHFFMVGF APLTSRGGSS YRQVNVPELT QQMFDPKNMM

PENIIT_KGO72779 RFPGQLNSDL RKLAVNMVPF PRLHFFMVGF APLTSRGGSS YRQVSVPELT QQMFDPKNMM

PYRPBR_KC342227 RFPGQLNSDL RKLAVNMVPF PRLHFFMVGF APLTSRGAHS FRAVTVPELT QQMFDPKNMM

RHYNSE_X81046 RFPGQLNSDL RKLAVNMVPF PRLHFFMVGF APLTSRGAHS FRAVTVPELT QQMFDPKNMM

SCLEHO_KF765483 RFPGQLNSDL RKLAVNMVPF PRLHFFMVGF APLTSRGAHS FRAVTVPELT QQMYDPKNMM

SCLESC_XM_001594794 RFPGQLNSDL RKLAVNMVPF PRLHFFMVGF APLTSRGAHS FRAVTVPELT QQMYDPKNMM

VENTIN_M97951 RFPGQLNSDL RKLAVNMVPF PRLHFFMVGF APLTSRGAHS FRAVTVPELT QQMFDPKNMM

....|....| ....|....| ....|....| ....|....| ....|....| ....|....|

305 315 325 335 345 355

ASPEND__M17519 AASDFRNGRY LTCSAIFRGK VSMKEVEDQM RNIQSKNQSY FVEWIPNNIQ SALCSIPPRG

BOTRCI_Z69263 AASDFRNGRY LTCSAIFRGK VSMKEVEDQM RNVQNKNSSY FVEWIPNNVQ TALCSIPPRG

CERCBE_AY856373 AASDFRNGRY LTCSAIYRGK VSMKEVEDQI RNVQNKNTAY FVEWIPNNVQ TALCSIPPRG

COCHHE_AB009971 AASDFRNGRY LTCSAYFRGK VSMKEVEDQM RNVQNKNSSY FVEWIPNNVQ TALCSIPPRG

GIBBFU_U27303 AASDFRNGRY LTCSAIFRGR VAMKEVEDQM RNVQNKNSSY FVEWIPNNIQ TALCAIPPRG

GIBBFU_KF771181 AAADFRNGRY LTCSAIFRGK LSTKEIEDQM LKVQTKNSEY FVDWIPNNVQ TSVCSVPPRG

GIBBZE_FJ214663 AAADFRNGRY LACSAIFRGR LSTKEIEDQM LKVQTKNSEY FVDWIPNNVQ TSVCSVPPRG

HELMSO_Y10670 AASDFRNGRY LTCCGIFRGK VSMKEVEDQM RNVQNKNSSY FVEWIPNNVQ TAICSIPPRG

HYPMOD_Y12256 AASDFRNGRY LTCCSIFRGK VAMKEVEDQM RNVQNKNSTY FVEWIPNNIQ TALCAIPPRG

LEPTNO_S56922 AASDFRNGRY LTCSAYFRGK VSMKEVEDQM RNVQNKNSSY FVEWIPNNVQ TALCSVPPRG

MONIFC_AY283676 AASDFRNGRY LTCSAIFRGK VSMKEVEDQM RNVQNKNSSY FVEWIPNNVQ TALCSIPPRG

MONILA_AY349149 AASDFRNGRY LTCSAILRGK VSMKEVEDQM RNVQNKNSSY FVEWIPNNVQ TALCSIPPRG

NEUSCR_M13630 AASDFRNGRY LTCSAIFRGK VSMKEVEDQM RNVQNKNSSY FVEWIPNNVQ TALCSIPPRG

PENIAU_JN112033 AASDFRNGRY LTCSALFRGK ISMKEVEDQM RNIQSKNQSY FVEWIPNNVQ TALCSVPPRG

PENIEX_KGO42666 AASDFRNGRY LTCSALFRGK ISMKEVEDQM RNIQNKNQSY FVEWIPNNVQ TALCSVPPRG

PENIIT_KGO72779 AASDFRNGRY LTCSALFRGK ISMKEVEDQM RNIQNKNQSY FVEWIPNNVQ TALCSVPPRG

PYRPBR_KC342227 AASDFRNGRY LTCSAIFRGK VSMKEVEDQM RNVQNKNSSY FVEWIPNNVQ TALCSIPPRG

RHYNSE_X81046 AASDFRNGRY LTCSAIFRGK VSMKEVEDQM RNVQNKNSSY FVEWIPTNVQ TALCSIPPRG

SCLEHO_KF765483 AASDFRNGRY LTCSAIFRGK VSMKEVEDQM RNVQNKNSSY FVEWIPNNVQ TALCSIPPRG

SCLESC_XM_001594794 AASDFRNGRY LTCSAIFRGK VSMKEVEDQM RNVQNKNSSY FVEWIPNNVQ TALCSIPPRG

VENTIN_M97951 AASDFRNGRY LTSSAIFRGK VSMKEVEDQM RNVQNKNSSY FVEWIPNNVQ TALCSIPPRG

**Beta-Tubulin Alignment (continued)**

....|....| ....|....| ....|....| ....|....| ....|....| ....|....|

365 375 385 395 405 415

ASPEND__M17519 LKMSSTFIGN STSIQELFKR VGDQFTAMFR RKAFLHWYTG EGMDEMEFTE AESNMNDLVS

BOTRCI_Z69263 LKMSSTFVGN STSIQELFKR VGDQFTAMFR RKAFLHWYTG EGMDEMEFTE AESNMNDLVS

CERCBE_AY856373 LKMSSTFVGN STSIQELFKR VGDQFTAMFR RKAFLHWYTG EGMDEMEFTE AESNMNDLVS

COCHHE_AB009971 LKMSSTFVGN STSIQELFKR VGDQFTAMFR RKAFLHWYTG EGMDEMEFTE AESNMNDLVS

GIBBFU_U27303 LTMSSTFIGN STSIQELFKR VGEQFTAMFR RKAFLHWYTG EGMDEMEFTE AESNMNDLVS

GIBBFU_KF771181 LPMAATFVGN STAIQEIFKR VDDQFSAMFR RKAFLHWYTS EGMDEMEFTE AQSNLHDLVS

GIBBZE_FJ214663 LDMSATFVGN STAVQEIFKR VDDQFSAMFR RKAFLHWYTS EGMDEMEFTE AQSNLHDLVS

HELMSO_Y10670 LKMSATFVGN STSIQELFKR VGDQFTAMFR RKAFLHWY-- ---------- ----------

HYPMOD_Y12256 LKMSSTFIGN STSIQELFKR VGDQFSAMFK RKAFLHWY-- ---------- ----------

LEPTNO_S56922 LKMSATFVGN STSIQELFKR IGDQFTAMFR RKAFLHWYTG EGMDEMEFTE AESNMNDLVS

MONIFC_AY283676 LKMSSTFVGN STSIQELFKR VGDQFTAMFR RKAFLHWY-- ---------- ----------

MONILA_AY349149 LKMSSTFVGN STSIQELFKR VGDQFTAMFR RKAFLHWY-- ---------- ----------

NEUSCR_M13630 LKMSSTFVGN STAIQELFKR IGEQFTAMFK RKAFLHWYTG EGMDEMEFTE AESNMNDLVS

PENIAU_JN112033 LKMSSTFVGN STSIQELFKR VGDQFTAMFR RK-------- ---------- ----------

PENIEX_KGO42666 LKMSSTFVGN STSIQELFKR VGDQFTAMFR RKAFLHWYTG EGMDEMEFTE AESNMNDLVS

PENIIT_KGO72779 LKMSSTFVGN STSIQELFKR IGDQFSAMFR RKAFLHWYTG EGMDEMEFTE AESNMNDLVS

PYRPBR_KC342227 LKMSSTFVGN STSIQELFKR IGDQFTAMFR RKAFLHWYTG EGMDEMEFTE AESNMNDLVS

RHYNSE_X81046 LKMSSTFVGN STSIQELFKR IGDQFTRMFR RKAFLHWYTG EGMDEMEFTE AESNMNDLVS

SCLEHO_KF765483 LKMSSTFVGN STSIQELFKR VGDQFTAMFR RKAFLHWYTG EGMDEMEFTE AESNMNDLVS

SCLESC_XM_001594794 LKMSSTFVGN STSIQELFKR VGDQFTAMFR RKAFLHWYTG EGMDEMEFTE AESNMNDLVS

VENTIN_M97951 LKMSSTFVGN STSIQELFKR VGDQFTAMFR RKAFLHWYTG EGMDEMEFTE AESNMNDLVS

....|....| ....|....| ....|...

425 435 445

ASPEND__M17519 EYQQYQDASI SEGEEEYAEE EIMEGEE-

BOTRCI_Z69263 EYQQYQDASI SEGEEEYEEE VPIEGEE-

CERCBE_AY856373 EYQQYQEASV SEGEEEYDEE APLEGEE-

COCHHE_AB009971 EYQQYQEASV SEGEEEYDEE APLEAEE-

GIBBFU_U27303 EYQQYQDAGI DEEEEEYEEE LP-EGEE-

GIBBFU_KF771181 EYQQYQDAEI DDEVEEYEGE GEPEEYEG

GIBBZE_FJ214663 EYQQYQDADI DDEAEEYE-E GEPEEYEG

HELMSO_Y10670 ---------- ---------- --------

HYPMOD_Y12256 ---------- ---------- --------

LEPTNO_S56922 EYQQYQEASI SEGEEEYDEE APLEAEE-

MONIFC_AY283676 ---------- ---------- --------

MONILA_AY349149 ---------- ---------- --------

NEUSCR_M13630 EYQQYQDAGV DEEEEEYEEE APLEGEE-

PENIAU_JN112033 ---------- ---------- --------

PENIEX_KGO42666 EYQQYQEASV SEGEEEYLAE DIVDEEV-

PENIIT_KGO72779 EYQQYQEASV SEGEEEYLAE DIVDEEV-

PYRPBR_KC342227 EYQQYQDASI SEGEEEYEEE AP------

RHYNSE_X81046 EYQQYQDASI SEGEEEYEEE APMEPEE-

SCLEHO_KF765483 EYQQYQDASV SEGEEEYEEE APIEGDE-

SCLESC_XM_001594794 EYQQYQDASI SEGEEEYEEE APIEGEE-

VENTIN_M97951 EYQQYQEASV SEGEEEYDEE APLEGEE-

**SdhB Alignment**

....|....| ....|....| ....|....| ....|....| ....|....| ....|....|

5 15 25 35 45 55

PYRNTE_XM_003302513 MACTRALTRL AT-KRIAVRP -AVFSRGFAS VNDVHARDPI SKTAEKIVPD AARPTIPESK

ALTEAL_KJ426262 MASIRAFTRL AT-QRTAVRP -AVFSRGFAS VNDVHARDPI SKTAEKIAPD ASRSPTPESK

ALTESO_KC517311 MASIRAFTRL ATSDLVPVRP -AVFSRGFAS VNDVHAREPI SKIAEKIAPD ASRSPVPESK

BOTRCI_AY726618 MAALRTGARS ARAIFAASRP --AFRTQMRT MASVDS---- -SVPESPTVS PSRPVESASK

CORYCA_AB548738 MACTRAFARL AT-TRTAVRP AAVFTRGFAS VTDTAAREPV SKVAEKIVPD PARKVVPESQ

DIDYBR_HQ156460 ---------- ---------- ---------- ---------- ---------- ----------

EUROOR_XM_001827434 MAALRSTSRL VASSKPLFRP -AVFARSYAT VDA------- ---------- ----------

PODOXA_AB547416 ---------- ---------- ---------- ---------- ---------- ----------

SCLESC_XM_001594527 MASLRTSARC ARSIFAASRP --AFRTQMRT MASVQSQ--- -TAVDSPTIS PSRPVEPASK

SEPTTR_XM_003850705 MA-----LRL AT---RRFAP -IAFRRGMAT TIE-HTKEPI SATAEALS-- ASRPPIKETK

USTIMA_XM_011388576 MSLFNVSNGL RT----ALRP SVASSSRVAA FSTTAA---- ---------- -ARLATPTS-

....|....| ....|....| ....|....| ....|....| ....|....| ....|....|

65 75 85 95 105 115

PYRNTE_XM_003302513 TSTVQEPSPS KDAKTKTFHI YRWNPDEPTS KPKMQSYTLD LNKTGPMMLD ALIRIKNEVD

ALTEAL_KJ426262 TSTIQEPEPS KDAKTKTFHI YRWNPDEPTS KPKMQSYTLD LNKTGPMMLD ALIRIKNEVD

ALTESO_KC517311 TSTVPEPEPS KDAKTKTFHI YRWNPDEPTS KPKMQSYTLD LNKTGPMMLD ALIRIKNEVD

BOTRCI_AY726618 TSTVKEPAAD SESLIKTFNI YRWNPDEPTS KPRMQSYTLD LNKTGPMMLD ALIRIKNEVD

CORYCA_AB548738 TSTVKDPQPD KDAKTKTFHI YRWNPDEPTS KPKMQTYTLD LNKTGPMMLD ALIRIKNELD

DIDYBR_HQ156460 ---------- ---------- ---------- ---------- ---------- ----------

EUROOR_XM_001827434 --AAQDPNPS ETPRTKTFHI YRWNPDQPTE KPKMQSYSLD LNKTGPMMLD ALIRIKNEMD

PODOXA_AB547416 ---------- ---------- ---------- ---------- ---------- ----------

SCLESC_XM_001594527 TSTVKEPETD SNSLIKTFHI YRWNPDEPAS KPRMQSYTLD LNKTGPMMLD ALIRIKNEVD

SEPTTR_XM_003850705 TSTVKEPQMD ADAKTKTFHI YRWNPDQPTD KPRMQSYTLD LNKTGPMMLD ALIRIKNEVD

USTIMA_XM_011388576 -DNVGS--SG KPQHLKQFKI YRWNPDKPSE KPRLQSYTLD LNQTGPMVLD ALIKIKNEID

....|....| ....|....| ....|....| ....|....| ....|....| ....|....|

125 135 145 155 165 175

PYRNTE_XM_003302513 PTLTFRRSCR EGICGSCAMN IDGVNTLACL CRIPTDTTKE SRIYPLPHMY VVKDLVPDMT

ALTEAL_KJ426262 PTLTFRRSCR EGICGSCAMN IDGVNTLACL CRIPTDTTKE SRIYPLPHMY VVKDLVPDMT

ALTESO_KC517311 PTLTFRRSCR EGICGSCAMN IDGVNTLACL CRIPTDTTKE SRIYPLPHMY VVKDLVPDMT

BOTRCI_AY726618 PTLTFRRSCR EGICGSCAMN IDGVNTLACL CRIPRDAKHE TKIYPLPHTY VVKDIVPDLT

CORYCA_AB548738 PTLTFRRSCR EGICGSCAMN IDGVNTLACL CRIPTDTTKE SRIYPLPHMY IVKDLVPDMT

DIDYBR_HQ156460 ---------- ------CAMN IDGVNTLACL CRIPTDTAKE SRIYPLPHMY VVKDLVPDMT

EUROOR_XM_001827434 PTLTFRRSCR EGICGSCAMN IDGVNTLACL CRIPTDTAKE SRIYPLPHTY VVKDLVPDLT

PODOXA_AB547416 ---------- ---------- ---------- ---------- ---------- ----------

SCLESC_XM_001594527 PTLTFRRSCR EGICGSCAMN IDGVNTLACL CRIPRDAKHE TKIYPLPHTY VVKDIVPDLT

SEPTTR_XM_003850705 PTLTFRRSCR EGICGSCAMN IDGVNTLACL CRIPTDTAKE TRIYPLPHTY VVKDLVPDMT

USTIMA_XM_011388576 PTLTFRRSCR EGICGSCAMN IDGVNTLACL CRI--DKQND TKIYPLPHMY IVKDLVPDLT

....|....| ....|....| ....|....| ....|....| ....|....| ....|....|

185 195 205 215 225 235

PYRNTE_XM_003302513 LFYKQYRSVK PYLQRTTAAP DGREFRQSKE DRKKLDGLYE CILCACCSTS CPSYWWNQEE

ALTEAL_KJ426262 LFYKQYRSVK PYLQRTTAAP DGREFRQSKE DRKKLDGLYE CILCACCSTS CPSYWWNQEE

ALTESO_KC517311 LFYKQYRSVK PYLQRSTAAP DGREFRQSKE DRKKLDGLYE CILCACCSTS CPSYWWNQEE

BOTRCI_AY726618 QFYKQYKSIK PYLQHTDPAP EGKEYLQSKE DRKKLDGLYE CILCACCSTS CPSYWWNSEE

CORYCA_AB548738 LFYKQYRSVK PYLQRDTPAP DGREYRQSKE ERKKLDGLYE CILCACCSTS CPSYWWNQEE

DIDYBR_HQ156460 LFYKQYRSVK PYLQRTTPSP DGREYRQTKE DRRKLDGLYE CILCACCSTS CPSYWWNQEE

EUROOR_XM_001827434 QFYKQYKSIK PYLQRETKTE DGLEYRQSPE ERKKLDGLYE CILCACCSTS CPSYWWNSEE

PODOXA_AB547416 ---------- ---------- ---------- ---------- ---------- CPSYWWNSEE

SCLESC_XM_001594527 QFYKQYKSIK PYLQHTDPAP GGKEYLQSKE ERKKLDGLYE CILCACCSTS CPSYWWNSEE

SEPTTR_XM_003850705 QFYKQYKSIK PYLQRDTAPP DGKENRQSVA DRKKLDGLYE CILCACCSTS CPSYWWNSEE

USTIMA_XM_011388576 QFYKQYRSIE PFLKSNNTPS EG-EHLQSPE ERRRLDGLYE CILCACCSTS CPSYWWNQDE

....|....| ....|....| ....|....| ....|....| ....|....| ....|....|

245 255 265 275 285 295

PYRNTE_XM_003302513 YLGPAVLLQS YRWIADSRDE KKAERQDALN NSMSLYRCHT ILNCSRTCPK GLNPALAIAE

ALTEAL_KJ426262 YLGPAVLLQS YRWIADSRDE KKAERQDALN NSMSLYRCHT ILNCSRTCPK GLNPALAIAE

ALTESO_KC517311 YLGPAVLLQS YRWIADSRDE KKAERQDALN NSMSLYRCRT ILNCSRTCPK GLNPALAIAE

BOTRCI_AY726618 YLGPAILLQS YRWLADSRDQ KKEERKAALD NSMSLYRCHT ILNCSRTCPK GLNPGLAIAE

CORYCA_AB548738 YLGPAVLLQS YRWIADSRDE KTAQRQDALN NSMSMYRCHT ILNCSRTCPK GLNPALAIAE

DIDYBR_HQ156460 YLGPAVLLQS YRWIADSRDE KKAERQDALN NSMSLYRCHT ILNCSRTC-- ----------

EUROOR_XM_001827434 YLGPAILLQS YRWLADSRDE KTAERKHALD NSMSVYRCHT ILNCSRTCPK GLNPARAIAE

PODOXA_AB547416 YLGPAVLMQS YRWLADSRDE KTEERKSALD NSMSLYRCHT ILNCTRTC-- ----------

SCLESC_XM_001594527 YLGPAILLQS YRWLADSRDQ KKEERKAALD NSMSLYRCHT ILNCSRTCPK GLNPGLAIAE

SEPTTR_XM_003850705 YLGPAVLLQS YRWINDSRDE KTAQRKDALN NSMSLYRCHT ILNCSRTCPK GLNPALAIAE

USTIMA_XM_011388576 YLGPAVLMQA YRWMADSRDD FGEERRQKLE NTFSLYRCHT IMNCSRTCPK NLNPGKAIAQ

**SdhB Alignment (continued)**

....|....| ....|....| .

305 315

PYRNTE_XM_003302513 IKKSMAFT-- ---------- -

ALTEAL_KJ426262 IKKSMAFT-- ---------- -

ALTESO_KC517311 IKKSMAFT-- ---------- -

BOTRCI_AY726618 IKKEMAF--- ---------- -

CORYCA_AB548738 IKKSMAFT-- ---------- -

DIDYBR_HQ156460 ---------- ---------- -

EUROOR_XM_001827434 IKKLMAAH-- ---------- -

PODOXA_AB547416 ---------- ---------- -

SCLESC_XM_001594527 IKKEMAF--- ---------- -

SEPTTR_XM_003850705 IKKSMAFTG- ---------- -

USTIMA_XM_011388576 IKKDMAVGAP KASERPIMAS S

**SdhC Alignment**

....|....| ....|....| ....|....| ....|....| ....|....| ....|....|

5 15 25 35 45 55

PYRNTE_XM_003302752 MASQRIFQLG LRRVAA--PS LRVQ------ --PA----G- -RLMQRRL-- AATGNASQSE

ALTEAL_KJ426267 MASQRVFQLG LRRAAA--PS LRVQ------ --PA----G- -RMVQRRL-- AATEHASQSE

BOTRCI_EMR82724 MFSQRATQQS LRRLAAGQPS LISQLAMRKL AAPAA--IG- -ASMQTRP-- VATQKLTPKD

CORYCA_AB548741 MASQRVFQLG LRRAAA--PS FKVQ------ --PA----G- -RMVQRRA-- AATQQVNESQ

EUROOR_XM_001824837 MISQKVAQQS LRRLAVQQPY AMRWSLMNS- ATPAAVAMG- -RFMQTRQ-- -AATTSNTSD

SCLESC_XM_001597417 MFSQRATQQS LRRLAAGQPS LISQLAMRRL AAPAA--IG- -ASMQTRP-- VTTQKLTPKD

SEPTTR_XM_003850403 MLAQKLTQQS LRRLALQ-PS TLRF------ ATPAAIALGN NSFQQQRRQV TAAAVSESHA

....|....| ....|....| ....|....| ....|....| ....|....| ....|....|

65 75 85 95 105 115

PYRNTE_XM_003302752 AAQILAKQRL NRPVSPHLAI YRPQITWLAS SLNRITGIVL SGSLYLFGIA YLVAPYTGWH

ALTEAL_KJ426267 AAEILAKQRV NRPVSPHLAI YKPQITWYAS SLNRITGITL SGSLYLFGIA YLIAPYTGWH

BOTRCI_EMR82724 SYNILVEQRK LRPVAPHLTI YQPQIPWIMS GLNRITGCIL SGGFYVFGAA YLASPLFGWH

CORYCA_AB548741 AQEILAKQRI QRPVSPHLSI YRPQITWYAS SFNRITGVAL SGGLYLFGFA YLAAPTLGWH

EUROOR_XM_001824837 PTKILAQQRL NRPVSPHLSI YRPQITWIGS SFHRITGFAL SGSLYLYATA YLASPLLGWH

SCLESC_XM_001597417 SYNILVEQRK LRPTSPHLTI YQPQIPWIMS GLFRITGCVL SGGFYVFGAA YLVSPLFGWH

SEPTTR_XM_003850403 RNEILAKQRL NRPVAPHLAI YKPQITWYLS ALNRVTGVAA SGAFYAFGLL YLAAPSLGWH

....|....| ....|....| ....|....| ....|....| ....|....| ....|....|

125 135 145 155 165 175

PYRNTE_XM_003302752 LETQSMVATV AAWPAAVKAG LKAFYAFPFF FHSLNGLRHL AWDVGVGFKN QQVIRTGWGV

ALTEAL_KJ426267 LETQSMVATV AAWPAAVKAG LKAFYALPFF FHSFNGLRHL AWDVGIGFKX QQVIRTGWTA

BOTRCI_EMR82724 LDTASMVAAF GAWPLAAKFL AKFTLAMPFT YHSFNGLRHL AWDMGKTFKN ATVVKTGWTV

CORYCA_AB548741 LETQSMVAAV AAWPVAAKVA AKISIAMPFF FHSLNGLRHL SWDIGLGFKN KAVIQTGWSV

EUROOR_XM_001824837 LESASVAAAF AALPIVAKVL LKGFMALPFT YHCFNGVRHL VWDLGRGITN QQVIKSGWTV

SCLESC_XM_001597417 LDTASMVAAF GAWPLAAKVL AKFSVALPFT YHSFNGLRHF SWDMGKTFKN ATVVKTGWAV

SEPTTR_XM_003850403 LESAALAASF GAWPVLLQVL TKTILALPVT FHSLNGVRHL VWDTASMITN KQVQTTGWTV

....|....| ....|

185 195

PYRNTE_XM_003302752 VGLTAVMGLY YTFAG

ALTEAL_KJ426267 VGLTVAFSLY YTFLG

BOTRCI_EMR82724 VGLSVGSALA LVAFL

CORYCA_AB548741 IALSAAATLY YSLFV

EUROOR_XM_001824837 VGLSVLSALA LAFL-

SCLESC_XM_001597417 IGLSVSSALA LVAFM

SEPTTR_XM_003850403 VGLSVASALG LAFL-

**SdhD Alignment**

....|....| ....|....| ....|....| ....|....| ....|....| ....|....|

5 15 25 35 45 55

PYRNTE_XM_003297196 MAS-SLRPG- LFSQVCAKPQ TSQRMLSTST TSTINRPLAQ QVRPAFQRSV IQQSTRIAAF

ALTEAL_FJ437068 MAS-VMRPG- LLRQACPPVQ QSQRMLSTAT -STMNRPLVQ QLRPAFQRSA IQKSTRIAAF

ALTESO_KC517316 MAS-VMRPG- LLRQACPPAH ASQRMLSTAT -ATTSRPLAQ QLRPAFQRSA VPKATRIAAF

BOTRCI_GQ253440 MAS-FIKPS- VIRQTC--LA ASKRNFSTKI PSSFPAINKP AGRSTFVRDA LPGSMRVAAF

CORYCA_AB548745 -----MK--- ---------R TS-------- ------PIAQ QLRPALERSQ APSATRIAAF

EUROOR_AB449815 MAS-IARQSS LLRQSC---- ---------- --------LS AFRSPFATRN GAGVSQVVAF

SCLESC_XM_001593201 MAS-LIKPS- MLRQTC--LA ASKRSFSTKV SSSFPAINKP AVRSAFVRDA LPGSMRVAAF

SEPTTR_JF916694 MASTALRPA- ALRQLLT-AT TTKR------ --------AS TLPAASLLR- -TQFTQRSGF

....|....| ....|....| ....|....| ....|....| ....|....| ....|....|

65 75 85 95 105 115

PYRNTE_XM_003297196 HATQRNSILP PLPQKIIGTA NDPTPVPDPD YTHGSYHWSF ERIVSAGLIP LTVAPFAAGS

ALTEAL_FJ437068 HATQRNQILP PLPQKIIGTT NDPVPVPDPD YAHGSYHWSF ERIVSAGLIP LTIAPFAAGS

ALTESO_KC517316 HATQRTQILP PLPQKIIGTT NDPVPVPDPD YAHGSYHWSF ERIVSAGLIP LTIAPFAAGS

BOTRCI_GQ253440 HASGRQSILP PLPQSIDGTS NDAAAVPKPS PSHGSYHWTF ERLIAVGLVP LTVAPFVSGS

CORYCA_AB548745 HATQRQQILP PLPQKIEGTL NDPARVPDPS PSHGSYHWSF ERAISAGLIP LTIAPFAAGS

EUROOR_AB449815 HASAKKQILP PLPQTIQGTM NDPAPIPTPH PSEGSYHWTF ERAISAGLVP LTIAPFAAGS

SCLESC_XM_001593201 HASGRQSILP PLPQSIDGTS NDAAPVPKPS PSHGSYHWTF ERLIAVGLIP LTVAPFVSGS

SEPTTR_JF916694 QTTARRPILP PLPQVIRGGV NDPAPVKEPS PSHGSYHWTM ERLVSAALIP LTIVPFAAGS

....|....| ....|....| ....|....| ....|....| ....|....| ....|....|

125 135 145 155 165 175

PYRNTE_XM_003297196 LNPVTDSILC ALLVVHSHIG FESCIVDYFP KKRVPKTRAA AMWALRAGTV VLGLALYSFE

ALTEAL_FJ437068 LNPLTDSILC ALLVVHSHIG FESCIIDYFP SKRVPKTRTA AMWALRAGTV ALGLALYSFE

ALTESO_KC517316 LNPLTDSILC ALLVVRSHIG FESCIIDYFP AKRVPKTRKA AMWALRAGTL TLGLALYSFE

BOTRCI_GQ253440 LNPATDALLC AAILIHSHIG FESCITDYFP SKRVPKTKAF LWWGLRGATV LVGVGLYEFE

CORYCA_AB548745 LNPVTDSILC ALLVIHSHIV FEACVIDYFP AKRIPAVRKA ANWALRIGTV TLGFALYSFE

EUROOR_AB449815 LNPVMDAVLC SFIVLHSHIG FQAAIIEYFP TRRVPKTATF CNWLLRAFTL TTAVGLYEFE

SCLESC_XM_001593201 LNPATDAILC AAILIHSHIG FESCVIDYIP RKRLPKTRAL FWWGLRGATV LVGVGLYEFE

SEPTTR_JF916694 LNPVLDGTFI GMIIIHSYIG FQSAITDYFP SWRVPKTRKL ADWANVAAVF LVGWGWYEFE

....|....| ....|....| ....|....| .

185 195 205

PYRNTE_XM_003297196 TNDVGITEAV ARLWHA---- ---------- -

ALTEAL_FJ437068 TNDVGITEAV ARLWHA---- ---------- -

ALTESO_KC517316 TNDVGITE-- ---------- ---------- -

BOTRCI_GQ253440 TNDVGVTEGI KRIWRA---- ---------- -

CORYCA_AB548745 TNDVGITEAV SQLWHA---- ---------- -

EUROOR_AB449815 TNDVGVTEAF KRVWKA---- ---------- -

SCLESC_XM_001593201 TNDVGLTEGI KRIWHA---- ---------- -

SEPTTR_JF916694 TNDIGLTAGI ARVWTAGATA KDAKNKIEQK L

**CesA3 Alignment**

....|....| ....|....| ....|....| ....|....| ....|....| ....|....|

5 15 25 35 45 55

PHYTIN_EF563995 MGLTGAGIIA SVVGILGGVS LSCGGWSSLS LGARSLFVTT QFLSAFAMGF VVAFSAIVSL

PHYTCP_JX905357 MGLTGAGIIA SVVGILGGIS LSCGGWSSLS LGARSLFVTT QFLSAFAMGF VVAFTAIVSL

PLASVI_GQ258975 MGLTGAGVIA SVIGILGGVS LSCGGWSSLS LGARSLFVTT QFLSAFAMGF VVAFTAIVSL

PSPECU_JF799098 MSLTGAGVIA SVVGILGGLS LSCGGWSSLS LGARSLFVTT QFVSAFAMGF VIAFTAIVSL

....|....| ....|....| ....|....| ....|....| ....|....| ....|....|

65 75 85 95 105 115

PHYTIN_EF563995 SDTNEWVAVA AGGGAGFVIA LIGGFMTIFG PYILILITGG LIACYLLLVD AYDGINVFPA

PHYTCP_JX905357 SDTNEWVAVA AGGGAGFVIA LIVGFMTFFG PYILILVTGG IISCYLLLID AYDGVNLFPA

PLASVI_GQ258975 SDTNEWVAVI AGGGAGFVIA LIVGFLTFFG PYILILITGG IIASYLLLID AFNGINVFPA

PSPECU_JF799098 SATNEWVALA AGGGAGFIVA LIVGFLTFFG PYILVLVTGS IIAAYLLLFD AHNGINVFPA

....|....| ....|....| ....|....| ....|....| ....|....| ....|....|

125 135 145 155 165 175

PHYTIN_EF563995 DNQLARQEFV IAFMIIFELV CCSSSKTSEL ENHRFKYIVF SAITGGWMAA DGVSRLIDSG

PHYTCP_JX905357 DNQLARQEFV IAFMIIFELV CCSSSKTSEL ENHRFKYIIF SAITGGWMAS DGVSRLIDSE

PLASVI_GQ258975 DNQLARQEFV IAFMIIFELV CSSTSKTSEL ENHRFKYIIF SCITGGWMAA DGLSRLIDSS

PSPECU_JF799098 DNQLARQEFV IAFMIIFELV CSSCSKTTEL ENHRFKYVLF SAITGGWLAA DGISRLIDSG

....|....| ....|....| ....|....| ....|....| ....|....| ....|....|

185 195 205 215 225 235

PHYTIN_EF563995 AVLSTVAYTS IQDGGKAAMD GIDASAQTLM FVIWGAVFVV GGLNQLSMRW GLMCYNRVGA

PHYTCP_JX905357 AVLSNVAFTS LQDGGKAALK GMDSGSQTLM FVIWGAVFVV GGLNQLSMRW GLMCYNRVGA

PLASVI_GQ258975 AVLSTVAFDS IQDGGKAALK GIDAGGQSLM FLLWAAVVVI GGLNQLSMRW GLLCYNRVGA

PSPECU_JF799098 AVLSDVAFQS IQEGGAAAVR GMDAGSQTLM FVVWGAIFLV GGLNQLAMRW GLGCYNRVGT

....|....| ....|....| ....|....| ....|....| ....|....| ....|....|

245 255 265 275 285 295

PHYTIN_EF563995 HAQLGPVEEQ MPELPTGATL PAQTMTERVR LVCENCFATV PAGTAFCTEC GEAMPSEDGN

PHYTCP_JX905357 HAQLGPVEEQ MPELPTGATL PAQAMTERVR LVCENCFATV PSGTAFCTEC GEAMPSDDAN

PLASVI_GQ258975 HAQLGPVEEQ LPELPTGATL PAQTITERVR LVCENCFATV PAGTAFCTEC GEAMPSEDAN

PSPECU_JF799098 HAQLGPVEEQ LPELPTGATL PAQVPTERVR LVCENCFATV PSGTAFCTEC GEAMPTDDAT

....|....| ....|....| ....|....| ....|....| ....|....| ....|....|

305 315 325 335 345 355

PHYTIN_EF563995 PDVSISQAQM PSVSMNNKGQ VPDRWQHVPH RTYMSTTSFV DPKHAKEGGV SMKDNGRSIR

PHYTCP_JX905357 PDVSISQAQM PSVTMNSKSQ VPDRWQQVPH RTFLSTTSFV DPKHAKEGGV SMKDNSRSIR

PLASVI_GQ258975 PDVSISQAQM PSVAMNNKSQ VPDRWQQVPH RTYMSTTSFV DPKHAKEGGV SMKDNSRSIR

PSPECU_JF799098 PEVSISQAQM PSVTMNNKSQ APDRWQQVPH RTFLSTTSFV DPKAAKEGGV SMKDNGRSIR

....|....| ....|....| ....|....| ....|....| ....|....| ....|....|

365 375 385 395 405 415

PHYTIN_EF563995 FMDSGVQGPD GKMSQYNDSI AGVRNYYEPS FRSFAMSTYS IANRAAEPVE TPNIRKYKMS

PHYTCP_JX905357 FMDSGVQGPD GKMSQYNDSI AGARNYYEPS FRSFAMSTYS IANRAAEPVE TPNIRKYKMS

PLASVI_GQ258975 FMDSGVQGPD GKMSQYNDSI AGVRNYYEPS FRSFAMSTYS IANRAAEPVE TPNIRKYKMS

PSPECU_JF799098 FMDSGVQGPD GKMSQYNDSI AGVRNYYEPS FRSFAMSTYS MANRAAEPVE TPNIRKYKMS

....|....| ....|....| ....|....| ....|....| ....|....| ....|....|

425 435 445 455 465 475

PHYTIN_EF563995 GSGMFHVFYF GTAATGIFWL YYLTTMYPQQ YFCDHARPTL PCSGLPTSET TGCYSSTVNF

PHYTCP_JX905357 GSGMFHVFYF GTAATGIFWL YYLTTMYPQQ YFCDHARPTL PCSELPTSET TGCYSSTVNF

PLASVI_GQ258975 GSGMFHVFYF GTAATGIFWL YYLTTMYPQQ YFCDHARPTL PCSELPTSET AGCYSSTVNF

PSPECU_JF799098 GSGMFHVFYF GTAATGVVWL YYLTTMYPQE YFCDHARPTL PCSSLPPSEV LGCYSSTVNF

....|....| ....|....| ....|....| ....|....| ....|....| ....|....|

485 495 505 515 525 535

PHYTIN_EF563995 DADSGDGYCI KDVPFMSWLM YAMMIFSEFL NYFLGLLFNF SMWRPIRRGA RYMNDFKPPI

PHYTCP_JX905357 DASSGDGYCI KDVPFMSWLM YAMMVFSEFL NYFLGLLFNF SMWRPIRRGA RYMNDFKPPI

PLASVI_GQ258975 DSASGDGYCI KDVPFMSWVM YAMMIFSEFL NFFLGLLFNF SMWRPIRRGA RFMNDFKPPI

PSPECU_JF799098 DANSGDGYCI QNVPFMSWVM YGMMIFSEFL NFFLGLLFNF SMWRPIRRGA RYMNDFKPPI

....|....| ....|....| ....|....| ....|....| ....|....| ....|....|

545 555 565 575 585 595

PHYTIN_EF563995 PKEQWPTVDI FLCHYMEPVT DSMQTLKNCL AMQYPPELLH IFILDDGYTK SVWDANNHFK

PHYTCP_JX905357 PKEQWPSVDI FLCHYMEPVT DSMQTLKNCL AMQYPPELLH IFILDDGYTK SVWDANNHFK

PLASVI_GQ258975 PKEQWPTVDI FLCHYMEPVT DSMQTLKNCL AMQYPPELLH IFILDDGYTK SVWDANNHFK

PSPECU_JF799098 PKEQWPTVDI FLCHYMEPVT DSMQTLKNCL AMQYPPELLH IFVLDDGYTK SVWDANNHFK

**CesA3 Alignment (continued)**

....|....| ....|....| ....|....| ....|....| ....|....| ....|....|

605 615 625 635 645 655

PHYTIN_EF563995 VTVNTKVIEV AGDLRGDLAR LMHERVVGPV QDDQSLKSWR RQHSSVRELR KEGGKGVQRR

PHYTCP_JX905357 VSVNTKVIEI CGDLRGDLAR LMHERVVGPV QDDQSLKTWR RQHSSVRELR KEGGKGVQRR

PLASVI_GQ258975 VTVNTKVIEI AGDLRGDLAR LMHERVVGPV QDDQSLKSWR RQHSSVRELR KEGGKGVQRR

PSPECU_JF799098 VTVNTKVIEI CGDLRGDLAR LMHERVVGPV QDDQSLKSWR RQHSSVRELR KEGGKGVQRR

....|....| ....|....| ....|....| ....|....| ....|....| ....|....|

665 675 685 695 705 715

PHYTIN_EF563995 DCAVGSLSDD YDYRDRGIPR VTFIGRMKPE THHSKAGNIN NALFNEGADG KYLLILDNDM

PHYTCP_JX905357 DCAVGSLSDD YDYRDRGIPR VTFIGRMKPE THHSKAGNIN NALFNEGADG KYLLILDNDM

PLASVI_GQ258975 DCAVGSLSDD YDYRDRGIPR VTFIGRMKPE THHSKAGNIN NALFNEGADG KYLLILDNDM

PSPECU_JF799098 DCAVGSLSDD YDYRDRGIPR VTFIGRMKPE THHSKAGNIN NALFNEGADG KYLLILDNDM

....|....| ....|....| ....|....| ....|....| ....|....| ....|....|

725 735 745 755 765 775

PHYTIN_EF563995 KPHPKFLLAV LPFFFSEGEA VDGGGRQYSD DISWNQVSYV QTPQYFEDTP QLTIMGDPCG

PHYTCP_JX905357 KPHPKFLLAV LPFFFSEGEA VDGGGRQYSD DISWNQVSYV QTPQYFEDTP QLTIMGDPCG

PLASVI_GQ258975 KPHPKFLLAV LPFFFSEGEA VDGGGRQYSD DISWNQVSYV QTPQYFEDTP QLTIMGDPCG

PSPECU_JF799098 KPHPKFLLAV LPFFFSEGEA VDGGGRQYSD DISWNQVAYV QTPQYFEDTP QLTIMGDPCG

....|....| ....|....| ....|....| ....|....| ....|....| ....|....|

785 795 805 815 825 835

PHYTIN_EF563995 HKNTIFFDAV QCGRDGFDSA AFAGTNAVFR RQAFDSIGGI CYGTQTEDAY TGNVLHTSGW

PHYTCP_JX905357 HKNTIFFDAV QCGRDGFDSA AFAGTNAVFR RQAFDSIGGI QYGTQTEDAY TGNVLHTSGW

PLASVI_GQ258975 HKNTIFFDAV QCGRDGFDSA AFAGTNAVFR RQAFDSIGGI CYGTQTEDAY TGNVLHTSGW

PSPECU_JF799098 HKNTIFFDAV QCGRDGFDSA AFAGTNAVFR RQAFDSIGGI QYGTQTEDAY TGNILHTSGW

....|....| ....|....| ....|....| ....|....| ....|....| ....|....|

845 855 865 875 885 895

PHYTIN_EF563995 DSVYFRKDFE GDAKDRIRLC EGAVPDTVAA AMGQKKRWAK GAVQILLMKN ESEVDPDWRP

PHYTCP_JX905357 DSVYFRKDFE GDAKDRIRLC EGAVPETVAA AMGQKKRWAK GAVQILLMKN ESEVDPDWRP

PLASVI_GQ258975 DSVYFRKDFE GDAKDRIRLC EGAVPETVAA AMGQKKRWAK GAVQILLMKN ESEVDPDWRP

PSPECU_JF799098 DSVYFRKDFE GDAKDRIRLC EGAVPETVAA AMGQKKRWAK GAVQILLMKN ESEVDPDWRP

....|....| ....|....| ....|....| ....|....| ....|....| ....|....|

905 915 925 935 945 955

PHYTIN_EF563995 PRVPAPDPKP SLTFPRKMFF YDSVLYPLGS IPALCYVSIA VYYLCTGDAP IYARGTKFLY

PHYTCP_JX905357 PRVPAPDPKP SLAFPRKMFF YDSVLYPFGS IPALCYVAIA VYYLCTGDAP IYARGTKFLY

PLASVI_GQ258975 PRVPAPDPKP SLTFPRKMFF YDSVLYPFGS IPALCYVSIA VYYLCTGDAP IYARGTKFLY

PSPECU_JF799098 PRVPAPDPKP SLAFPRKMFF YDSVLYPFGS IPALCYVSIA VYYLCTGDAP IYARGTKFLY

....|....| ....|....| ....|....| ....|....| ....|....| ....|....|

965 975 985 995 1005 1015

PHYTIN_EF563995 SFLPVTFCRW VLNLLANRAV DNNDVWRAQQ TWFSFSFITM MAIVEAIQAR VTGKDKSWAN

PHYTCP_JX905357 SFLPVTFCRW VLNLLANRAV DNNDVWRAQQ TWFSFSFITM MAIVEAIQAR MTGKDKSWAN

PLASVI_GQ258975 SFLPVTFCRW VLNLLANRAV DNNDVWRAQQ TWFSFSFITM MAIVEAIQAR VTGKDKSWAN

PSPECU_JF799098 SFLPVTFCRW VLNLLANRAV DNNDVWRAQQ TWFSFSFITM MAIVEAIQAR MTGKDKSWAN

....|....| ....|....| ....|....| ....|....| ....|....| ....|....|

1025 1035 1045 1055 1065 1075

PHYTIN_EF563995 TGAGQKTSWT EIPNVLFFFT LLFSQLVALV RFFEYENATN PWNYVSAMFF GFFVMSQFYP

PHYTCP_JX905357 TGAGQKTSWT EIPNVLFFFT LLFSQLVALI RFFEYENATN PWNYVSAMFF GFFVMSQFYP

PLASVI_GQ258975 TGAGQKTSWT EIPNVLFFFT LLFSQLVALI RFFEYENATN PWNYVSAMFF GFFVMSQFYP

PSPECU_JF799098 TGAGQKTSWT EIPNVLFFFT LLFSQLVALI RFFEYENATN PWNYVSAMFF GFFVMSQFYP

....|....| ....|....| ....|....| ....|....| ....|....| ....|....|

1085 1095 1105 1115 1125 1135

PHYTIN_EF563995 MVKMSITEYC GWDHTAATFT ANVFGSLLVV YIVVFVQLWQ VYYEGNLQVA QGTDAGGSAA

PHYTCP_JX905357 MVKMSITEYC GWDHTAATFT ANVFGSLLVV YIVVFVQLWQ VYYEGNLQVA QGTDAGSTSS

PLASVI_GQ258975 MVKMSITEYC GWDHTAATFT ANVFGSLLVV YIVVFVQLWQ VYYEGNLLVA QGTDGGGNAE

PSPECU_JF799098 MVKMSITEYC GWDHTAATFT ANVFGSLLVV YIVVFVQLWQ VYYAGNLLVA QGADGGEAAT

....|....

1145

PHYTIN_EF563995 AT-------

PHYTCP_JX905357 ---------

PLASVI_GQ258975 TGDAATTVT

PSPECU_JF799098 TTTT-----

**Os-1 Alignment**

....|....| ....|....| ....|....| ....|....| ....|....| ....|....|

5 15 25 35 45 55

BOTRCI_AF435964 MEDSTIAHTT AILQTLALSS IDLP----LT NVYGN----- -KGIRLPGAD TAEKLALERE

ALTEBI_AY700092 MAAETYSSVS AIIRNLARQH DPTRDPSFSA QVSANGAKTA VNAIALPGPE SDEKTQLQQE

ALTELO_DQ887538 MAAETYSSVS AIIRNLARQH DPTRDPSFSA QVSANGAKTA ANAIALPGPE SEEKTQLEQE

PLEOAL_EU711371 MAAETYSGVS AIIRNLARQH DPARDPSFSA QVSANGAKTA ANQIALPGPE SEEKTQLEQE

....|....| ....|....| ....|....| ....|....| ....|....| ....|....|

65 75 85 95 105 115

BOTRCI_AF435964 LAALVSRVQR LEARAITVNN Q------TLP DTPNELGAPS AFADVLTG-- -APSRASK-S

ALTEBI_AY700092 LSALCSRIDF LEHKSNHAAN QQGQFPLTPA QEPSDEGALY TPAGVFRGNN GPPAQGRRGS

ALTELO_DQ887538 LSALCSRIDF LEHKSSHAPN QQGQFPLTPA QEPPEEGALY TPAGVFRGNS GPPAQGRRGS

PLEOAL_EU711371 LLALCSRIDF LEHKSSNAAG KSGQFPLTPA QEPPEEGVLY TQAGAIRNSN GPPGQARRGS

....|....| ....|....| ....|....| ....|....| ....|....| ....|....|

125 135 145 155 165 175

BOTRCI_AF435964 TTSRQQLVNS LLAAREAPTG GERPPKFTKL SDEELEALRE HVDHQSKQLD SQKSELAGVH

ALTEBI_AY700092 NKERAIWVSN WLAAKESNGD PEQP--AAAL TEEQLNYLRV HLNQQADQIR NQREHIDNLS

ALTELO_DQ887538 NKERAIWVSN WLAAKESNGD PEQP--AAAL TEEQLNYLRV HLNQQADQIR NQREHIDNLS

PLEOAL_EU711371 NKERATWVSN WLAAKESNGG PEEP--AVAL TEEQFNYLRV HLHQQADQIR NQREHIDNLS

....|....| ....|....| ....|....| ....|....| ....|....| ....|....|

185 195 205 215 225 235

BOTRCI_AF435964 AQLFEQKQRQ EQALNVLEVE RVAALERELK KHQQANEAFQ KALREIGEIV TAVARGDLSK

ALTEBI_AY700092 QEVNKQLTTQ SMVF-EHGIE DIGALKRELG KHQQANLAFQ KALREIGAIV TAVAMGDLSK

ALTELO_DQ887538 QEVNKQLTTQ SMVF-EHGIE DIGALKRELG KHQQANLAFQ KALREIGAIV TAVAMGDLSK

PLEOAL_EU711371 QEVNKQLTTQ SMVF-EHGIE DIGALKRELG KHQQANLAFQ KALREIGAIV TAVAMGDLSK

....|....| ....|....| ....|....| ....|....| ....|....| ....|....|

245 255 265 275 285 295

BOTRCI_AF435964 KVQIHSVEMD PEITTFKRVI NTMMDQLQIF SSEVSRVARE VGTEGILGGQ AKISGVDGTW

ALTEBI_AY700092 KVLIHAKEMD PEITLFKRTI NTMVDQLQEF ASQVTFLARE VGTEGRLGGQ ANLPGVAGIW

ALTELO_DQ887538 KVLIHAKEMD PEITLFKRTI NTMVDQLQEF ASQVTFLARE VGTEGRLGGQ ANLPGVAGIW

PLEOAL_EU711371 KVLIHAKEMD PEITLFKRTI NTMVDQLQEF ASQVTFLARE VGTEGRLGGQ ANLPGVAGIW

....|....| ....|....| ....|....| ....|....| ....|....| ....|....|

305 315 325 335 345 355

BOTRCI_AF435964 KELTDNVNVM AQNLTDQVRE IASVTTAVAH GDLTQKIERP AQGEILQLQQ TINTMVDQLR

ALTEBI_AY700092 AELTD-INGM AKNLTDQVRE IAVVTTAVAM GDLSRKIERP ARGEILQLQQ TINSMVDQLQ

ALTELO_DQ887538 AELTDSVNGM AKNLTDQVRE IAVVTTAVAM GDLSRKIERP ARGEILQLQQ TINSMVDQLQ

PLEOAL_EU711371 AELTD-INMM ANNLTEQVRE IAVVTTAVAH GDLSRKIERP ARGEILQLQQ TINTMVDQLQ

....|....| ....|....| ....|....| ....|....| ....|....| ....|....|

365 375 385 395 405 415

BOTRCI_AF435964 TFAAEVTRVA RDVGTEGILG GQAEIEGVQG MWNTLIVNVN AMANNLTTQV RDIAIVTTAV

ALTEBI_AY700092 SFATQVTKVA RDVGTEGKLG GQAEIAGVKG MWNELTVNVN AMAQNLTTQV RDIAQVTTAV

ALTELO_DQ887538 SFATQVTKVA RDVGTEGKLG GQAEIAGVKG MWNELTVNVN AMAQNLTTQV RDIAQVTTAV

PLEOAL_EU711371 SFATEVTKVA RDVGTEGKLG GQAEIAGVKG MWNELTVNVN AMAQNLTTQV RDIAQVTTAV

....|....| ....|....| ....|....| ....|....| ....|....| ....|....|

425 435 445 455 465 475

BOTRCI_AF435964 AKGDLTQKVQ AECKGEIKQL KETINSMVDQ LQQFAREVTK IAREVGTEGR LGGQATVHDV

ALTEBI_AY700092 AQGNLTRKVE AECKGEILEL KNTINRMVDQ LQQFAHEVTK IAREVGSEGR LGGQATVHGV

ALTELO_DQ887538 AQGNLTRKVE AECKGEILEL KNTINRMVDQ LQQFAHEVTK IAREVGSEGR LGGQATVHGV

PLEOAL_EU711371 AQGNLTRKVE AQCKGEILEL KNTINRMVDQ LQQFAHEVTK IAREVGSEGR LGGQATVHGV

....|....| ....|....| ....|....| ....|....| ....|....| ....|....|

485 495 505 515 525 535

BOTRCI_AF435964 EGTWRDLTEN VNGMAMNLTT QVREIAKVTT AVARGDLTKK IEVEVQGEIA SLKDTINTMV

ALTEBI_AY700092 EGTWKDLTEN VNGMAMNLTT QVREIAEVTT AVARGDLSRK VKAEVQGEIL SLKITINTMV

ALTELO_DQ887538 EGTWKDLTEN VNGMAMNLTT QVREIAEVTT AVARGDLSRK VKAEVQGEIL SLKITINTMV

PLEOAL_EU711371 EGTWKDLTEN VNGMAMNLTT QVREIAEVTT AVARGDLSRK VKAEVQGEIL SLKITINTMV

....|....| ....|....| ....|....| ....|....| ....|....| ....|....|

545 555 565 575 585 595

BOTRCI_AF435964 DRLSTFAFEV SKVAREVGTD GTLGGQAQVD NVEGKWKDLT ENVNTMARNL TTQVRGISTV

ALTEBI_AY700092 DRLNTFAQEV SKVAREVGTD GILGGQAQVD NVEGKWKDLT NNVNTMAQNL TLQVRSISEV

ALTELO_DQ887538 DRLNTFAQEV SKVAREVGTD GILGGQAQVD NVEGKWKDLT NNVNTMAQNL TLQVRSISEV

PLEOAL_EU711371 DRLNTFAQEV SKVAREVGTD GILGGQAQVD NVEGKWKDLT NNVNTMAQNL TLQVRSISEV

**Os-1 Alignment (continued)**

....|....| ....|....| ....|....| ....|....| ....|....| ....|....|

605 615 625 635 645 655

BOTRCI_AF435964 TQAIANGDMS QKIEVAAAGE ILILKETINN MVDRLSIFSN EVQRVAKDVG VDGKMGGQAD

ALTEBI_AY700092 TQAIAKGDMS RRVHVDAEGE IRLLKDTVND MVMRLDEWSL AVKRVARDVG VDGKMGGQAD

ALTELO_DQ887538 TQAIAKGDMS RRVHVDAEGE IRLLKDTVND MVMRLDEWSL AVKRVARDVG VDGKMGGQAD

PLEOAL_EU711371 TQAIAKGDMS RRVHVDAEGE IRLLKDTVND MVMRLDEWSL AVKRVARDVG VDGKMGGQAD

....|....| ....|....| ....|....| ....|....| ....|....| ....|....|

665 675 685 695 705 715

BOTRCI_AF435964 VAGIGGRWKE ITTDVNTMAN NLTTQVRAFG DITNAATDGD FTKLITVEAS GEMDELKRKI

ALTEBI_AY700092 VRDIDGRWKE ITTDVNTMAQ NLTSQVRAFG DITNAAMEGK FT-QITVEAS GEMDELKRKI

ALTELO_DQ887538 VRDIDGRWKE ITTDVNTMAQ NLTSQVRAFG DITNAAMEGK FT-QITVEAS GEMDELKRKI

PLEOAL_EU711371 VRDIDGRWKE ITTDVNTMAQ NLTSQVRAFG DITNAAMEGK FT-QITVEAS GEMDELKRKI

....|....| ....|....| ....|....| ....|....| ....|....| ....|....|

725 735 745 755 765 775

BOTRCI_AF435964 NQMVYNLRDS IQRNTLAREA AEFANRTKSE FLANMSHEIR TPMNGIIGMT QLTLDTDLTQ

ALTEBI_AY700092 NQMVSSLRES IQRNTAAREA AELANKTKSE FLANMSHEIR TPMNGIIGMT QLTLDTDLTH

ALTELO_DQ887538 NQMVSSLRES IQRNTAAREA AELANKTKSE FLANMSHEIR TPMNGIIGMT QLTLDTDLTH

PLEOAL_EU711371 NQMVSSLRES IQRNTAAREA AELANKTKSE FLANMSHEIR TPMNGIIGMT QLTLDTDLTH

....|....| ....|....| ....|....| ....|....| ....|....| ....|....|

785 795 805 815 825 835

BOTRCI_AF435964 YQREMLNIVH NLANSLLTII DDILDLSKIE ANRMIMEEIP YTLRGTVFNA LKTLAVKANE

ALTEBI_AY700092 AQREMLTIVH NLAGQLLTII DDILDISKIE ANRMVMEEIP FSMRGTIFNA LKSSASRANE

ALTELO_DQ887538 AQREMLTIVH NLAGQLLTII DDILDISKIE ANRMVMEEIP FSMRGTIFNA LKSLASRANE

PLEOAL_EU711371 AQREMLTIVH NLAGQLLTII DDILDISKIE ANRMVMEEIP FSMRGTIFNA LKSLASRANE

....|....| ....|....| ....|....| ....|....| ....|....| ....|....|

845 855 865 875 885 895

BOTRCI_AF435964 KFLDLTYRVD SSVPDHVVGD SFRLRQVILN LVGNAIKFTE HGEVSLTIQK AEQDHCAPNE

ALTEBI_AY700092 RKLNLAYDVS YKVPDYVVGD SFRLRQIILN LVGNAIKLTE PGEVKVAISM AQEQECDPDH

ALTELO_DQ887538 RKLNLAYDVS YKVPDYVVGD SFRLRQIILN LVGNAIKFTE LGEVKVAISM AQEQECDPDH

PLEOAL_EU711371 RKLNLAYDVS YTVPDYVVGD SFRLRQIILN LVGNAIKFTE HGEVKVAISM APEQECGPDH

....|....| ....|....| ....|....| ....|....| ....|....| ....|....|

905 915 925 935 945 955

BOTRCI_AF435964 YAVEFCVSDT GIGIQADKLN LIFDTFQQAD GSMTRKFGGT GLGLSISKRL VNLMRGDVWV

ALTEBI_AY700092 YVFQFAVSDT GIGIRGDKLN LIFDTFQQAD GSTTRKFGGT GLGLSISKRL VTLMGGRMWV

ALTELO_DQ887538 YVFQFAVSDT GIGIRGDKLN LIFDTFQQAD GSTTRKFGGT GLGLSISKRL VTLMGGRMWV

PLEOAL_EU711371 YVFQFAVSDT GIGIRGDKLN LIFDTFQQAD GSTTRKFGGT GLGLSISKRL VTLMGGRMWV

....|....| ....|....| ....|....| ....|....| ....|....| ....|....|

965 975 985 995 1005 1015

BOTRCI_AF435964 KSQYGKGSSF YFTCTVRLAT SDISFIQKQL KPYQGHNVLF IDKGQTGHGK EIITMLTQLG

ALTEBI_AY700092 ESDAGKGSVF YFTYKVRLGK PAIQAIQPQL VAYKQHTVLF VDQGNTGFSD EITEHLKTLD

ALTELO_DQ887538 ESDVGKGSVF YFTCKVRLGK PAIQAIQPQL VAYKQHTVLF VDQGNTGFSD QIIEHLKTLD

PLEOAL_EU711371 ESDFGKGSVF YFTLTVRLGK PEITAIQPQL LHNKQHTVLF VDQRNTGFSD QIIEHLKALD

....|....| ....|....| ....|....| ....|....| ....|....| ....|....|

1025 1035 1045 1055 1065 1075

BOTRCI_AF435964 LVPVVVDSEQ HTILLGNGRT KEKIASTYDV IVVDSIESAR KLRSIDEFKY IPIVLLAPVI

ALTEBI_AY700092 LVPMVVHSVE EVP-----ET TKRTDMPYDC VIVDNDKTAR ELRIAERFKY IPLVMLTPHV

ALTELO_DQ887538 LVPMVVHSVE EVP-----ET TKRTDMPYDC VIVDNDKTAR ELRIAERFKY IPLVMLTPHV

PLEOAL_EU711371 LVPMVVNSVE EVP-----ET TKRADMPYDC VIVDNDKTAR ELRIAERFKY IPLVMLTPHV

....|....| ....|....| ....|....| ....|....| ....|....| ....|....|

1085 1095 1105 1115 1125 1135

BOTRCI_AF435964 HVSLKSALDL GITSYMTTPC LTIDLGNGMI PALENRAAPS LADNTKSFDI LLAEDNIVNQ

ALTEBI_AY700092 CISLRSALEN GISSYMTTPC LPIDLGNALI PALDGRAAPL VSDHSKSFQI LLAEDNAVNQ

ALTELO_DQ887538 CISLRSALEN GISSYMTTPC LPIDLGNALI PALDGRAAPL VSDHSKSFQI LLAEDNAVNQ

PLEOAL_EU711371 CISLRSALEN GISRYMTTPC LPIDLGNALI PALDGRAAPL VSDHSKSFQI LLAEDNAVNQ

....|....| ....|....| ....|....| ....|....| ....|....| ....|....|

1145 1155 1165 1175 1185 1195

BOTRCI_AF435964 RLAVKILEKY HHVVTVVGNG QEALDAIKEK RYDVILMDVQ MPIMGGFEAT AKIREYERSL

ALTEBI_AY700092 KLAVRILEKY HHRVTVANNG LEAFEHIQKK RYDCVLMDVQ MPVMGGFEAT AKIREWERDN

ALTELO_DQ887538 KLAVRILEKY HHRVTVANNG LEAFEHIQKK RYDCVLMDVQ MPVMGGFEAT AKIREWERDN

PLEOAL_EU711371 KLAVRILEKY HHRVTVANNG LEAYEHIQKK RYDCVLMDVQ MPVMGGFEAT AKIREWEREN

**Os-1 Alignment (continued)**

....|....| ....|....| ....|....| ....|....| ....|....| ....|....|

1205 1215 1225 1235 1245 1255

BOTRCI_AF435964 GTQRTPIIAL TAHAMLGDRE KCIQAQMDEY LSKPLKQNHL IQTILKCATL GGALLEKGRE

ALTEBI_AY700092 GIPSTPVIAL TAHAMVGDRE KCLAAQMDDY LSKPLRQNQL IQTILRCATV GSTMYDHSSE

ALTELO_DQ887538 GIPSTPVIAL TAHAMVGDRE KCLAAQMDDY LSKPLRQNQL IQTILRCATV GSTMYDHSSE

PLEOAL_EU711371 GIPSTPVIAL TAHAMVGDRE KCLAAQMDDY LSKPLRQNQL IQTILRCATV GSTIYDHTSE

....|....| ....|....| ....|....| ....|....| ....|....| ....|....|

1265 1275 1285 1295 1305 1315

BOTRCI_AF435964 VRQSANEESP ------NSQN GPRGAQHPAS SPTPAHMRPA IEPRAYTTTG PINHGSAESP

ALTEBI_AY700092 PRYTASSHLP NITLPGKGND NGGASSSSGG LPSPGKKRPQ LEARGFTE-- --RGG-AQSP

ALTELO_DQ887538 PRYTASSHLP NIALPAKGND NGGASSSSGG LPSPGKKRPQ LDTRGFTE-- --RGGGAQSP

PLEOAL_EU711371 PRYTASSHIP KLDMPGNSKE KSSAGASSSS AAPSPKKRPQ LETRGFTE-- --RGGGAESP

....|....| ....|....| ...

1325 1335

BOTRCI_AF435964 SLVTADAEDP --LARLLMRA HSS

ALTEBI_AY700092 NLLAVDQTDN GSVERLLLRS HSS

ALTELO_DQ887538 NLLAVDQTDN GSVERLLLRS HSS

PLEOAL_EU711371 NLLAVDQTDN GSVERVRSLS ASL

**Cyp51B Alignment**

....|....| ....|....| ....|....| ....|....| ....|....|

10 20 30 40 50

SEPTTR - AY253234 MGLLQEVLAQ FDAQFG---- -------QTS LWKLVGLGFL AFSTLAILLN

ASPEFL - KOC13803 MGILAVILDS VCERCS---- -------GSS LWMLSTVALL SILVVSVVIN

CANDAL - AF153850 ----MAIVET VID------- -----GINYF LSLSVTQQIS ILLGVPFVYN

CERCBE - HM778021 MGLLQDVAVA FDNHFG---- -------QTA TWKLVLLAST TFFLLSVVVN

ERYSGH - AJ578761 MGISESFMFP YLQPLL---- -------QLG FGIALASGIL SLLLLLTFLN

ERYSGT - AJ578751 MGKPESFMSP YLQPLL---- -------QFG FSIALASGII SLLLLLTFLN

FILBNF - AF225914 ---MSAIIPQ VQQLLGQVAQ FFPPWFAALP TSLKVAIAVV GIPALIIGLN

MONIFC - AF470621 MGVLETIAGP LAQEIS---- -------QRS TGTIIAAGVA AFVVLAVVLN

MYCOFI - XP_007928752 MGLLQDAAAL FDAQFG---- -------QTA TWKLVPLGFS IFFAVSVLLN

PHAKPA - KC741475 ---MSSSV-- IIDQLY---- -------SFS TTSLIITSLT SILTIIVILN

PUCCRT - FJ976683 ---MSSVIGS LLEPIG---- -------SFS TFNQVLIYLV LAVVSIISIN

SACCCE - AAB68433 MSATKSIVGE ALEYVNI--- -----GLSHF LALPLAQRIS LIIIIPFIYN

UNCINE - AAC49812 MYIADILSDL LTQQTT---- -------RYG WIFMVTSIAF SIILLAVGLN

....|....| ....|....| ....|....| ....|....| ....|....|

60 70 80 90 100

SEPTTR - AY253234 VLSQLLFRGK SSDPPLVFHW VPFIGSTITY GIDPYKFFFS CREKYGDVFT

ASPEFL - KOC13803 VLRQLLFKNY -KEPPLVFHW FPFIGSTISY GMDPYRFFFN CREKYGDIFT

CANDAL - AF153850 LVWQYLYSLR KDRAPLVFYW IPWFGSAASY GQQPYEFFES CRQKYGDVFS

CERCBE - HM778021 VLRQVLFRQP KNEPPLVFHF VPIIGSTISY GIDPYKFFFS CREKYGDVFT

ERYSGH - AJ578761 VLKQLLFKNP -NEPPIVFHW IPIIGSTISY GMNPYKFFHE SQAKYGNIFT

ERYSGT - AJ578751 VLKQLLFKNP -NEPPIVFHW IPIIGSTISY GMNPYKFFHE SQAKYGNIFT

FILBNF - AF225914 VFQQLCLPRR KDLPPVVFHY IPWFGSAAYY GEDPYKFLFE CRDKYGDLFT

MONIFC - AF470621 VLNQVLFANP -NEPPVVFHW LPIIGSTITY GIDPYRFFFD CRAKYGDVFT

MYCOFI - XP_007928752 VLRQLLFRNP -NEPPLVFHY VPFIGSTISY GIDPYKFFFA CRQKYGDCFT

PHAKPA - KC741475 VTNQLLF-KD RNTPPLVFHI FPVLGSVISY GMDPYQFFED CRRKHGNVFT

PUCCRT - FJ976683 IFDQLAIPKD PTAPPVVFHL FPFIGSAVSY GIDPYAFLES CRKKYGNVFT

SACCCE - AAB68433 IVWQLLYSLR KDRPPLVFYW IPWVGSAVVY GMKPYEFFEE CQKKYGDIFS

UNCINE - AAC49812 VLSQLLFRRP -YEPPVVFHW FPIIGSTISY GIDPYKFYFD CRAKYGDIFT

....|....| ....|....| ....|....| ....|....| ....|....|

110 120 130 140 150

SEPTTR - AY253234 FILLGKKTTV CLGTKGNDFI LNGKLKDVNA EEIYSPLTTP VFGKDVVYDC

ASPEFL - KOC13803 FVLLGKKTTV YLGTKGNDFI LNGKLRDVCA EEVYSPLTTP VFGRHVVYDC

CANDAL - AF153850 FMLLGKIMTV YLGPKGHEFV FNAKLSDVSA EDAYKHLTTP VFGKGVIYDC

CERCBE - HM778021 FILLGKKTTV CLGTKGNDFI LNGKLRDVNA EEIYSPLTTP VFGKDVVYDC

ERYSGH - AJ578761 FILLGKKTTV YLGRQGNNFI LNGKLRDVNA EEIYTVLTTP VFGTDVVYDC

ERYSGT - AJ578751 FILLGKKTTV YLGRQGNNFI LNGKLRDVNA EEVYSVLTTP VFGTDVVYDC

FILBNF - AF225914 FILMGRRITV ALGPKGNNLS LGGKISQVSA EEAYTHLTTP VFGKGVVYDC

MONIFC - AF470621 FILLGKKTTV YLGRKGNDFI LNGKHKDLNA EEIYTVLTTP VFGKDVVYDC

MYCOFI - XP_007928752 FILLGKKTTV VLGTKGNVFI LNGKLKDVNA EEIYSPLTTP VFGTDVVYDC

PHAKPA - KC741475 FVLLNKKVTV ALGPEGNTLV LNGKLSEVNA EEAYTHFTTP VFGKDVVYDV

PUCCRT - FJ976683 FVLLNKKVTV ALGLEGNALV LNGKLSQVNA EEAYTALTTP VFGTDVVYDV

SACCCE - AAB68433 FVLLGRVMTV YLGPKGHEFV FNAKLADVSA EAAYAHLTTP VFGKGVIYDC

UNCINE - AAC49812 FILLGKKVTV YLGLQGNNFI LNGKLKDVNA EEIYTNLTTP VFGRDVVYDC

....|....| ....|....| ....|....| ....|....| ....|....|

160 170 180 190 200

SEPTTR - AY253234 PNSKLMEQKK FVKYGLTTSA LQSYVTLIAA ETRQFFDRNN PHKKFASTSG

ASPEFL - KOC13803 PNAKLMEQKK FVKFGLTSDA LRSYVRLITE EVEDFVQKSS ---ALQGPNG

CANDAL - AF153850 PNSRLMEQKK FAKFALTTDS FKRYVPKIRE YILNYFVTDE SFKLKEKTHG

CERCBE - HM778021 PNSKLMEQKK FVKFGLTSAA LQSYVTLITE ETRQFFSKNN PHKRFASTSG

ERYSGH - AJ578761 PNSKLMEQKK FMKAALTTEA FRSYVPIIQN EVKSFIEKCD ---DFRKSKG

ERYSGT - AJ578751 PNSKLMEQKK FMKAALTTEA FRSYVPIIQN EVESFINKCD ---DFRKSEG

FILBNF - AF225914 PNEMLMQQKK FIKSGLTTES LQSYPPMITS ECEDFFTKEV GI-SPQKPSA

MONIFC - AF470621 PNAKLMEQKK FMKIGLSTEA FRSYVPIIQM EVENFMKRSS ---AFKGQKG

MYCOFI - XP_007928752 PNSKLMEQKK FVKYGLTSSA LQSYVKLITK ETKDFFSKDN PSKKFASTHG

PHAKPA - KC741475 PNSILMQQKK FIKAGLTTEK FKKYVGIIVR EATSYLEDHL FC-SPNVKSV

PUCCRT - FJ976683 PNAILMQQKK FVKSGLTNEN FRKYVSLIAE ETISYLEDHV FE-NPKTQQT

SACCCE - AAB68433 PNSRLMEQKK FVKGALTKEA FKSYVPLIAE EVYKYFRDSK NFRLNERTTG

UNCINE - AAC49812 PNSKLMEQKK FMKTALTIEA FHSYVTIIQN EVEAYINNCV ---SFQGESG

**Cyp51B Alignment (continued)**

....|....| ....|....| ....|....| ....|....| ....|....|

210 220 230 240 250

SEPTTR - AY253234 TIDLPPALAE LTIYTASRSL QGKEVREGFD SSFADLYHYL DMGFTPINFM

ASPEFL - KOC13803 VFDVCKTIAE ITIYTASRSL QGKEVRSRFD STFAELYHDL DMGFAPINFM

CANDAL - AF153850 VANVMKTQPE ITIFTASRSL FGDEMRRIFD RSFAQLYSDL DKGFTPINFV

CERCBE - HM778021 TLDLPPAFAE LTIYTASRSL QGKEVRSKFD STFAELYHYL DMGFTPINFM

ERYSGH - AJ578761 IINIDAVMAE ITIYTASHTL QGKEVRDRFD SSLAVLYHDL DMGFTPINFM

ERYSGT - AJ578751 IINIAAVMAE ITIYTASHTL QGKEVRDRFD SSLAVLYHDL DMGFTPINFM

FILBNF - AF225914 TLDLLKAMSE LIILTASRTL QGKEVRESLN GQFAKYYEDL DGGFTPLNFM

MONIFC - AF470621 TANIPPAMAE ITIYTASHTL QGKEVRDRFD TSFASLYHDL DMGFSPINFM

MYCOFI - XP_007928752 TVDLPPAMAE LTIYTASRSL QGKEVREKFD SSFADLYHDL DMGFTPINFM

PHAKPA - KC741475 TKDVHDITSE ITICTAAATL QGKEVREGLD KSFAKLYHDL DGGFTPLNFV

PUCCRT - FJ976683 VKDTFKVASE ITICTASATL QGPEVREALN KSFAQLYHDL DGGFTPLHFA

SACCCE - AAB68433 TIDVMVTQPE MTIFTASRSL LGKEMRAKLD TDFAYLYSDL DKGFTPINFV

UNCINE - AAC49812 TVNISKVMAE ITIYTASHAL QGEEVRENFD SSFAALYHDL DMGFTPINFT

....|....| ....|....| ....|....| ....|....| ....|....|

260 270 280 290 300

SEPTTR - AY253234 LPWAPLPQNR RRDYAQKKMS ETYMSIIQKR RES---KTGE HEEDMIHNLM

ASPEFL - KOC13803 LPWAPLPHNR KRDAAQKRMT ETYMEIIKER REAG---SKK DSEDMVWNLM

CANDAL - AF153850 FPNLPLPHYW RRDAAQKKIS ATYMKEIKSR RERG---DID PNRDLIDSLL

CERCBE - HM778021 LPWAPLPQNR RRDYAQKKMT ETYMSIIKQR REAGDKTSEN GEEDMIKNLM

ERYSGH - AJ578761 LHWAPLPHNR ARDHAQRTVA KIYMEIINSR RTQK--ETDD SNLDIMWQLM

ERYSGT - AJ578751 LHWAPLPHNR ARDHAQRTVA KIYMEIINSR RTQK--ETDN SNLDIMWQLM

FILBNF - AF225914 FPNLPLPSYK RRDEAQKAMS DFYLKIMENR RKGE---SDH EH-DMIENLQ

MONIFC - AF470621 LHWAPLPHNR ARDHAQRTVA STYMDIIQKR RAQA--TEAE FKSDIMWQLM

MYCOFI - XP_007928752 LPWAPLPQNR ARDRAQKKMA EVYTAIIKER REKGEPTSGE KEQDMIWNLM

PHAKPA - KC741475 FPNLPLPSYR RRDKAQVSMT NFYLNILKKR RAEN---RQD EFNDMLDVLQ

PUCCRT - FJ976683 FPNLPLPSYR RRDRAQLAMR NFYMNIIKKR REDD---REG QLGDMIDSLQ

SACCCE - AAB68433 FPNLPLEHYR KRDHAQKAIS GTYMSLIKER RKNN---DIQ -DRDLIDSLM

UNCINE - AAC49812 FYWAPLPWNR ARDHAQRTVA RTYMNIIQAR REEK--RSGE NKHDIMWELM

....|....| ....|....| ....|....| ....|....| ....|....|

310 320 330 340 350

SEPTTR - AY253234 Q-CKYKDGNA IPDKEIAHMM IALLMAGQHS SSATESWITL RLASRPDIQD

ASPEFL - KOC13803 S-CMYKDGTP VPDEEIAHMM IALLMAGQHS SSSTAAWIVL HLAASPEITE

CANDAL - AF153850 IHSTYKDGVK MTDQEIANLL IGILMGGQHT SASTSAWFLL HLGEKPHLQD

CERCBE - HM778021 S-CTYKDGTP IPDREVAHMM IALLMAGQHS SSSTSSWIFL RLATRRDIQD

ERYSGH - AJ578761 R-SSYKDGTP VPDKEIAHMM IALLMAGQHS SSSSSTWIML WLAARPDITE

ERYSGT - AJ578751 R-SSYKDGTP VPDKEIAHMM IALLMAGQHS SSSSSTWIML WLAARPDITE

FILBNF - AF225914 S-CKYRNGVP LSDRDIAHIM IALLMAGQHT SSATSSWTLL HLADRPDVVE

MONIFC - AF470621 R-SSYKDGTP VPDKEIANMM IALLMAGQHS SSSSISWIML RLAARPDIME

MYCOFI - XP_007928752 Q-CQYKNGQA IPDKEIAHMM IALLMAGQHS SSSTSCWILL RLASRPDIQD

PHAKPA - KC741475 ?-QHYKDGRA LSDREIAHIM IAVLMAGQHT SAATG?WLLT HLAHCPDLVD

PUCCRT - FJ976683 G-QTYKDGRP LNDKEIAHIM IALLMAGQHT SAATGSWLLL HLASRPDIVA

SACCCE - AAB68433 KNSTYKDGVK MTDQEIANLL IGVLMGGQHT SAATSAWILL HLAERPDVQQ

UNCINE - AAC49812 R-STYKDGTP VPDREIAHMM IALLMAGQHS SSSTSSWIML WLAARPDIME

....|....| ....|....| ....|....| ....|....| ....|....|

360 370 380 390 400

SEPTTR - AY253234 ELLQEQKDML GVNADGS--I KELTYANLSK LTLLNQVVKE TLRIHAPVHS

ASPEFL - KOC13803 ELYQEQLRIL GHD------M PPLTYENLQK LDLHAKVIKE TLRIHAPIHS

CANDAL - AF153850 VIYQEVVELL KEKG---GDL NDLTYEDLQK LPSVNNTIKE TLRMHMPLHS

CERCBE - HM778021 ELVQEQKDVL GVNEDGS--I KELTYENLSK LTLLSQVVKE TLRIHAPIHS

ERYSGH - AJ578761 ELYQEQLELL GSE------L PPLKYEDLSK LSLHQNVLKE VLRLHAPIHS

ERYSGT - AJ578751 ELYQEQLEIL GSE------L PPVKYEDLSK LTLHQNVVKE VLRLHAPIHS

FILBNF - AF225914 ALYQEQKQKL GNPDGTFR-- -DYRYEDLKE LPIMDSIIRE TLRMHAPIHS

MONIFC - AF470621 ELYQEQIEVL GAD------L PDLKYEDLSK LTLHQNILKE TLRLHTPIHS

MYCOFI - XP_007928752 ELLQEQKDVL GVNADGS--I KELTYADISR LPLLNQVVKE TLRLHAPIHS

PHAKPA - KC741475 RLRREQ?EVF GKGDGSG--E LEDLDYDRLQ TPLLNSCIKE VLRLHPPIHS

PUCCRT - FJ976683 ELRQEQIDLF GKPGQTDDQE LDPLDLERVQ SPLMIACIKE VLRLHPPIHS

SACCCE - AAB68433 ELYEEQMRVL D------GGK KELTYDLLQE MPLLNQTIKE TLRMHHPLHS

UNCINE - AAC49812 ELYEEQLRIF GSEK--P--F PPLQYEDLSK LQLHQNVLKE VLRLHAPIHS

**Cyp51B Alignment (continued)**

....|....| ....|....| ....|....| ....|....| ....|....|

410 420 430 440 450

SEPTTR - AY253234 ILRKVKSPMP IEG------- -TAYVIPTTH TLLAAPGTTS RMDEHFPDCL

ASPEFL - KOC13803 IIRAVKNPMP VEG------- -TPYVIPTSH NVLSSPGVTA RSEEHFPDPL

CANDAL - AF153850 IFRKVTNPLR IPE------- -TNYIVPKGH YVLVSPGYAH TSERYFDNPE

CERCBE - HM778021 ILRKVKSPMP VEG------- -TPYVIPTTH SLLAAPGATS RMDEHFPEPL

ERYSGH - AJ578761 ILRKVKNPMP VPG------- -TSYVIPKTH SLLAAPGWTS RDASYFPNPL

ERYSGT - AJ578751 ILRKVKNPMP VPG------- -TSYVIPKTH SLLAAPGWTS RDASYFPNPL

FILBNF - AF225914 IYRKVLSDIP VPPSLSAPSE NGQYIIPKGH YIMAAPGVSQ MDPRIWQDAK

MONIFC - AF470621 IMRKVTTPMP VSG------- -TKYVIPTSH TLMASPGCTS RDAEYFPEPL

MYCOFI - XP_007928752 ILRQVKSPMP LEG------- -TPYVVPTTH SLLAAPGATS RMDEHFPEAM

PHAKPA - KC741475 ILRKVKSPIL VPKTLSSIDK NNQYIIPSSH YVLAAPGVSQ IDPSVWDHPK

PUCCRT - FJ976683 IMRKVKSPIT VPRTLASRNE DTPYIIPSSN FVLAAPGTAQ LDGSIWSSPH

SACCCE - AAB68433 LFRKVMKDMH VPN------- -TSYVIPAGY HVLVSPGYTH LRDEYFPNAH

UNCINE - AAC49812 IMRKVKNPMI VPG------- -TKYVIPTSH VLISSPGCTS QDATFFPDPL

....|....| ....|....| ....|....| ....|....| ....|....|

460 470 480 490 500

SEPTTR - AY253234 HWEPHRWDES PSEKYKHLSP TTALGSIAEE KEDDGYGLVS KGAASPYLPF

ASPEFL - KOC13803 EWKPHRWDEA ---------- -IAVSSEDEE KVDYGYGLVT KGTNSPYLPF

CANDAL - AF153850 DFDPTRWDTA AA-------K ANSVSFNSSD EVDYGFGKVS KGVSSPYLPF

CERCBE - HM778021 LWEPHRWDES PDEKYAHLVP SMVKEAVAEE KEDYGYGLVS KGAASPYLPF

ERYSGH - AJ578761 KWDPHRWDTG SG-------- GVIGTDMEDE KFDYGYGLIS TGAASPYLPF

ERYSGT - AJ578751 KWDPHRWDTG SG-------- GVIGTDMEDE KFDYGYGLIS TGAASPYLPF

FILBNF - AF225914 VWNPARWHDE KG-----FAA AAMVQYTKAE QVDYGFGSVS KGTESPYQPF

MONIFC - AF470621 EWDPHRWDIG SG-------- RVIGNDQDEE FQDYGYGMIS KGASSPYLPF

MYCOFI - XP_007928752 LWEPHRWDEN PSEKYAHLAP KHVKEGVAEE TEDYGYGLVS KGAASPYLPF

PHAKPA - KC741475 EFRPERWLS- -------NFK KDKQ-EQEEE MVDYGFGAIS SGANSPYLPF

PUCCRT - FJ976683 EFDPSRWLK- -------LQS PFKAGETQEE MVDYGFGMIS SGANSPFLPF

SACCCE - AAB68433 QFNIHRWN-- ---------K DSASSYSVGE EVDYGFGAIS KGVSSPYLPF

UNCINE - AAC49812 KWDPHRWDIG SG-------- KVLGNDAVDE KYDYGYGLTS TGASSPYLPF

....|....| ....|....| ....|....| ....|....| ....|....|

510 520 530 540 550

SEPTTR - AY253234 GAGRHRCIGE QFAYVQLQTI TATMVRDFKF YNVDGSDNVV GTDYSSLFSR

ASPEFL - KOC13803 GAGRHRCIGE QFAYVQLGAI TAALVRLFKF SNLPGVQTLP DTDYSSLFSK

CANDAL - AF153850 GGGRHRCIGE QFAYVQLGTI LTTFVYNLRW TI--DGYKVP DPDYSSMVVL

CERCBE - HM778021 GAGRHRCIGE QFAYVQLQTI TATVLRDFKF YNPDGSKKVV DTDYSSLFSR

ERYSGH - AJ578761 GAGRHRCIGE QFATVQLVTI MATMVRSFKF HNLDGRNSVA ETDYSSMFSR

ERYSGT - AJ578751 GAGRHRCIGE QFATVQLVTI MATMVRSFKF HNLDGRNSVA ETDYSSMFSR

FILBNF - AF225914 GAGRHRCVGE QFAYTQLSTI FTYVVRNFTL KLA--VPKFP ETNYRTMIVQ

MONIFC - AF470621 GAGSHRCIGE QFANVQLITI MATVVRLFKF KNPDGSKDVI GTDYTSLFTG

MYCOFI - XP_007928752 GAGRHRCIGE QFAYVQLQTI TSEVIRDFKL YNVDGSDKVV GTDYSSLFSR

PHAKPA - KC741475 GAGRHRCIGE QFAYIQLAAV AVAVIRNCDL ELV--RKEFP LPDYTTMLVG

PUCCRT - FJ976683 GAGRHRCIGE QFAYIQLSTF AATVIRNCDL ELT--APEFP KPDYTTMLVC

SACCCE - AAB68433 GGGRHRCIGE HFAYCQLGVL MSIFIRTLKW HYP-EGKTVP PPDFTSMVTL

UNCINE - AAC49812 GAGRHRCIGE QFATLQLVTI MATMVRFFRF RNIDGKQGVV KTDYSSLFSM

....|....| ....|....|

560 570

SEPTTR - AY253234 PLSPAVVKWE RREEKEEKN-

ASPEFL - KOC13803 PLGNSKIQFE KREPVTKA--

CANDAL - AF153850 PTEPAEIIWE KRETCMF---

CERCBE - HM778021 PLSPAVVKWE RREKK-----

ERYSGH - AJ578761 PMAPATIAWE KRDKKDKTEC

ERYSGT - AJ578751 PMAPATIAWE KRDKKDKTEC

FILBNF - AF225914 PNNP-LVTFT LRNAEVKQEV

MONIFC - AF470621 PLEPAVVAWE RR--------

MYCOFI - XP_007928752 PLSPAVVRWE RREKK-----

PHAKPA - KC741475 PRKPTTVKFT RRN-------

PUCCRT - FJ976683 PLKPRDIKFT RRNHL-----

SACCCE - AAB68433 PTGPAKIIWE KRNPEQKI--

UNCINE - AAC49812 PLAPALIGWE KR--------

**Cyp51A Alignment**

....|....| ....|....| ....|....| ....|....| ....|....| ....|....|

5 15 25 35 45 55

ASPEFU_AF338659 ---------- -------MVP MLWLTAYMAV AVLTAILLNV VYQLFFRLWN RTEPPMVFHW

AJELCP_AAU01158 MGHLADVVAR VCAHCSTLSL WALLLAGSAT FIVLSVIINL LHQLLWK--N PNEPPVVFHW

ASPEFL_XM_002375082 ---------- -MIFSRSMAS FTLVSAYAAA GLLAIIVLNL LRQLLFR--N KTDPPLVFHW

ASPEPA_AY690428 ---------- -------MAS FTLVSAYAAA GLLAIIVLNL LRQLLFR--N KTDPPLVFHW

....|....| ....|....| ....|....| ....|....| ....|....| ....|....|

65 75 85 95 105 115

ASPEFU_AF338659 VPFLGSTISY GIDPYKFFFA CREKYGDIFT FILLGQKTTV YLGVQGNEFI LNGKLKDVNA

AJELCP_AAU01158 FPIIGSTISY GIDPYKFFLD CREKYGDIFT FVLLGKKTTV FLGTKGNDFI LNGKLKDVCA

ASPEFL_XM_002375082 IPFLGSTVTY GMDPYAFFFS CRQKYGDIFT FILLGRKITV YLGIQGNEFI LNGKLKDVNA

ASPEPA_AY690428 IPFLGSTVTY GMDPYAFFFS CRQKYGDIFT FILLGRKITV YLGIQGNEFI LNGKLKDVNA

....|....| ....|....| ....|....| ....|....| ....|....| ....|....|

125 135 145 155 165 175

ASPEFU_AF338659 EEVYSPLTTP VFGSDVVYDC PNSKLMEQKK FIKYGLTQSA LESHVPLIEK EVLDYLRDSP

AJELCP_AAU01158 EEVYSPLTTP VFGRHVVYDC PNSKLMEQKK FVKYGLTSES LRSYVTLITD EFNRYIKTSP

ASPEFL_XM_002375082 EEIYSPLTTP VFGSDIVYDC PNSKLMEQKK FIKFGLTQAA LESHVPLIEK EVLDYLKTSP

ASPEPA_AY690428 EEIYSPLTTP VFGSDIVYDC PNSKLMEQKK FIKFGLTQAA LESHVPLIEK EVLDYLKTSP

....|....| ....|....| ....|....| ....|....| ....|....| ....|....|

185 195 205 215 225 235

ASPEFU_AF338659 NFQGSSGRMD ISAAMAEITI FTAARALQGQ EVRSKLTAEF ADLYHDLDKG FTPINFMLPW

AJELCP_AAU01158 AFQGDKGVLD VCKSVSEITI YTASRSLQGK EVRSKFDSSF AQLYHDLDMG FTPINFMFPW

ASPEFL_XM_002375082 NFKGTSGRVE ITDAMAEITI FTAGRALQGE EVRKKLTAEF ADLYHDLDRG FTPINFMLPW

ASPEPA_AY690428 NFKGTSGRVE ITGAMAEITI FTAGRALQGE EVRKKLTAEF ADLYHDLDRG FTPINFMLPW

....|....| ....|....| ....|....| ....|....| ....|....| ....|....|

245 255 265 275 285 295

ASPEFU_AF338659 APLPHNKKRD AAHARMRSIY VDIINQRRLD GDKDSQKSDM IWNLMNCTYK NGQQVPDKEI

AJELCP_AAU01158 APLPHNRKRD AAQQKMSKIY TDIIRQRREA GVKKDS-EDM VWNLMSCVYK DGTPLPDIEI

ASPEFL_XM_002375082 APLPRNRKRD AAHARMREIY MDIINERRKN PDRET--SDM IWNLMHCTYK NGQPLPDKEI

ASPEPA_AY690428 APLPRNRKRD AAHVRMREIY MDIINERRKN SDRET--SDM IWNLMHCTYK NGQPVPDKEI

....|....| ....|....| ....|....| ....|....| ....|....| ....|....|

305 315 325 335 345 355

ASPEFU_AF338659 AHMMITLLMA GQHSSSSISA WIMLRLASQP KVLEELYQEQ LANLGPAGPD GSLPPLQYKD

AJELCP_AAU01158 AHMMIALLMA GQHSSSATLS WIILRLASCP HIIEELYEEQ KRVLG----- EDLPPLTYET

ASPEFL_XM_002375082 AHMMITLLMA GQHSSSSISS WIMLRLASEP AVMEELYQEQ ITKLSPDG-- RTLPPLQYRD

ASPEPA_AY690428 AHIMITLLMA GQHSSSSISS WIMLRLASEP AVMEELYQEQ ITKLSPDG-- RTLPPLQYRD

....|....| ....|....| ....|....| ....|....| ....|....| ....|....|

365 375 385 395 405 415

ASPEFU_AF338659 LDKLPFHQHV IRETLRIHSS IHSIMRKVKS PLPVPGTPYM IPPGRVLLAS PGVTALSDEH

AJELCP_AAU01158 LQNLNLNSHV IRETLRIHAP IHSILRAVKS PMPVDGTRYT IPTTHNLLAA PGVTSRLPEH

ASPEFL_XM_002375082 LDLLPLHQNL IKETLRLHLS IHSLMRKVKN PMPVPGTPYV VPADHVLLAS PGVTALSDEY

ASPEPA_AY690428 LDLLPLHQNL IKETLRLHLS IHSLMRKVKN PMPVPGTPYV VPADHVLLAS PGVTALSDEY

....|....| ....|....| ....|....| ....|....| ....|....| ....|....|

425 435 445 455 465 475

ASPEFU_AF338659 FPNAGCWDPH RWEN--QATK EQENDEVVDY GYGAVSKGTS SPYLPFGAGR HRCIGEKFAY

AJELCP_AAU01158 FPNPMTWDPH RWENPAMAQV EDQSDEKLDY GYGLVSKGAN SPYLPFGSGR HRCIGEQFAY

ASPEFL_XM_002375082 FPNASRWDPH RWEN--RVEK EDEED-IVDY GYGTVSKGTS SPYLPFGAGR HRCIGEKFAY

ASPEPA_AY690428 FPNASRWDPH RWEN--RVEK EDEED-TVDY GYGTVSKGTS SPYLPFGAGR HRCIGEKFAY

....|....| ....|....| ....|....| ....|....| ....|....| ....|..

485 495 505 515 525 535

ASPEFU_AF338659 VNLGVILATI VRHLRLFNVD GKKGVPETDY SSLFSGPMKP SIIGWEKRSK NTSK---

AJELCP_AAU01158 VQLGTLLVAI VRQLKLKKLD GETGVPETDY S--VRPPRDP FFWPDVSCFL TFGRLRC

ASPEFL_XM_002375082 VNLGVIVATM ARHMKLFNVD GKKGVPATDY SSMFSGPSKP AIIGWERRFP EKS----

ASPEPA_AY690428 VNLGVIVATM ARHMKLFNVD GKKGVPATDY SSMFSGPSKP AIIGWERRFP EKS----

**References for Tables and Figures**

1. Banno S, Fukumori F, Ichiishi A, Okada K, Uekusa H, Kimura M and Fujimura M, Genotyping of benzimidazole-resistant and dicarboximide-resistant mutations in Botrytis cinerea using real-time polymerase chain reaction assays. *Phytopathology*; **98**(4): 397-404 DOI Electronic Resource Number (2008).

2. Becher R and Wirsel SG, Fungal cytochrome P450 sterol 14α-demethylase (CYP51) and azole resistance in plant and human pathogens. *Applied microbiology and biotechnology*; **95**(4): 825-840 DOI Electronic Resource Number (2012).

3. Blum M, Gamper HA, Waldner M, Sierotzki H and Gisi U, The cellulose synthase 3 (CesA3) gene of oomycetes: structure, phylogeny and influence on sensitivity to carboxylic acid amide (CAA) fungicides. *Fungal biology*; **116**(4): 529-542 DOI Electronic Resource Number (2012).

4. Carter HE, Cools HJ, West JS, Shaw MW and Fraaije BA, Detection and molecular characterisation of Pyrenopeziza brassicae isolates resistant to methyl benzimidazole carbamates. *Pest management science*; **69**(9): 1040-1048 DOI Electronic Resource Number (2013).

5. Chen C-J, Yu J-J, Bi C-W, Zhang Y-N, Xu J-Q, Wang J-X and Zhou M-G, Mutations in a β-tubulin confer resistance of Gibberella zeae to benzimidazole fungicides. *Phytopathology*; **99**(12): 1403-1411 DOI Electronic Resource Number (2009).

6. Chen Z, Gao T, Liang S, Liu K, Zhou M and Chen C, Molecular mechanism of resistance of Fusarium fujikuroi to benzimidazole fungicides. *FEMS microbiology letters*; **357**(1): 77-84 DOI Electronic Resource Number (2014).

7. Cooley RN and Caten CE, Molecular analysis of the Septoria nodorum β-tubulin gene and characterization of a benomyl-resistance mutation. *Molecular and General Genetics MGG*; **237**(1-2): 58-64 DOI Electronic Resource Number (1993).

8. Fujimura M, Oeda K, Inoue H and Kato T, A single amino-acid substitution in the beta-tubulin gene of Neurospora confers both carbendazim resistance and diethofencarb sensitivity. *Current genetics*; **21**(4-5): 399-404 DOI Electronic Resource Number (1992).

9. Howard SJ and Arendrup MC, Acquired antifungal drug resistance in Aspergillus fumigatus: epidemiology and detection. *Medical mycology*; **49**(sup1): S90-S95 DOI Electronic Resource Number (2011).

10. Ishii H and Hollomon DW. *Fungicide Resistance in Plant Pathogens: Principles and a Guide to Practical Management*. Springer, (2015).

11. Jung MK and Oakley BR, Identification of an amino acid substitution in the benA, β‐tubulin gene of Aspergillus nidulans that confers thiabendazole resistance and benomyl supersensitivity. *Cell motility and the cytoskeleton*; **17**(2): 87-94 DOI Electronic Resource Number (1990).

12. Jung MK, Wilder IB and Oakley BR, Amino acid alterations in the benA (β‐tubulin) gene of Aspergillus nidulans that confer benomyl resistance. *Cell motility and the cytoskeleton*; **22**(3): 170-174 DOI Electronic Resource Number (1992).

13. Jung MK, May GS and Oakley BR, Mitosis in wild-type and β-tubulin mutant strains ofAspergillus nidulans. *Fungal Genetics and Biology*; **24**(1): 146-160 DOI Electronic Resource Number (1998).

14. Ma Z and Michailides TJ, Advances in understanding molecular mechanisms of fungicide resistance and molecular detection of resistant genotypes in phytopathogenic fungi. *Crop Protection*; **24**(10): 853-863 DOI Electronic Resource Number (2005).

15. Morio F, Loge C, Besse B, Hennequin C and Le Pape P, Screening for amino acid substitutions in the Candida albicans Erg11 protein of azole-susceptible and azole-resistant clinical isolates: new substitutions and a review of the literature. *Diagnostic microbiology and infectious disease*; **66**(4): 373-384 DOI Electronic Resource Number (2010).

16. Orbach MJ, Porro EB and Yanofsky C, Cloning and characterization of the gene for beta-tubulin from a benomyl-resistant mutant of Neurospora crassa and its use as a dominant selectable marker. *Molecular and Cellular Biology*; **6**(7): 2452-2461 DOI Electronic Resource Number (1986).

17. Pang Z, Shao J, Chen L, Lu X, Hu J, Qin Z and Liu X, Resistance to the novel fungicide pyrimorph in Phytophthora capsici: risk assessment and detection of point mutations in CesA3 that confer resistance. *PloS one*; **8**(2): 565 DOI Electronic Resource Number (2013).

18. Qiu J, Xu J, Yu J, Bi C, Chen C and Zhou M, Localisation of the benzimidazole fungicide binding site of Gibberella zeae β2‐tubulin studied by site‐directed mutagenesis. *Pest management science*; **67**(2): 191-198 DOI Electronic Resource Number (2011).

19. Qiu J, Huang T, Xu J, Bi C, Chen C and Zhou M, β‐Tubulins in Gibberella zeae: their characterization and contribution to carbendazim resistance. *Pest management science*; **68**(8): 1191-1198 DOI Electronic Resource Number (2012).

20. Quello KL, Chapman KS and Beckerman JL, In situ detection of benzimidazole resistance in field isolates of Venturia inaequalis in Indiana. *Plant Disease*; **94**(6): 744-750 DOI Electronic Resource Number (2010).

21. Sagatova AA, Keniya MV, Wilson RK, Monk BC and Tyndall JD, Structural insights into binding of the antifungal drug fluconazole to Saccharomyces cerevisiae lanosterol 14α-demethylase. *Antimicrobial agents and chemotherapy*; **59**(8): 4982-4989 DOI Electronic Resource Number (2015).

22. Stensvold CR, Jørgensen LN and Arendrup MC, Azole-resistant invasive aspergillosis: relationship to agriculture. *Current Fungal Infection Reports*; **6**(3): 178-191 DOI Electronic Resource Number (2012).

23. Trkulja N, Ivanović Ž, Pfaf-Dolovac E, Dolovac N, Mitrović M, Toševski I and Jović J, Characterisation of benzimidazole resistance of Cercospora beticola in Serbia using PCR-based detection of resistance-associated mutations of the β-tubulin gene. *European journal of plant pathology*; **135**(4): 889-902 DOI Electronic Resource Number (2013).

24. Yan K and Dickman MB, Isolation of a beta-tubulin gene from Fusarium moniliforme that confers cold-sensitive benomyl resistance. *Applied and environmental microbiology*; **62**(8): 3053-3056 DOI Electronic Resource Number (1996).

25. Yarden O and Katan T, Mutations leading to substitutions at amino acids 198 and 200 of beta-tubulin that correlate with benomyl-resistance phenotypes of field strains of Botrytis cinerea. *Phytopathology*; **83**(12): 1478-1483 DOI Electronic Resource Number (1993).

26. Yin Y, Liu X, Shi Z and Ma Z, A multiplex allele-specific PCR method for the detection of carbendazim-resistant Sclerotinia sclerotiorum. *Pesticide biochemistry and physiology*; **97**(1): 36-42 DOI Electronic Resource Number (2010).

27. Ziogas BN, Nikou D, Markoglou AN, Malandrakis AA and Vontas J, Identification of a novel point mutation in the β-tubulin gene of Botrytis cinerea and detection of benzimidazole resistance by a diagnostic PCR-RFLP assay. *European journal of plant pathology*; **125**(1): 97-107 DOI Electronic Resource Number (2009).
